# Schisandrin B confers multi-organ protection via regulation of mitochondrial homeostasis: mechanistic integration, organ-specific differences, and translational challenges—a review

**DOI:** 10.3389/fphar.2026.1781376

**Published:** 2026-03-27

**Authors:** Siyi Guo, Fei Yu, Chao Wang, Wenbing Zhao, Xuan Li, Jiayi Liu, Zhida Lyu

**Affiliations:** 1 Zibo Hospital of Traditional Chinese Medicine, Zibo, China; 2 Zibo Central Hospital, Zibo, China; 3 Shandong University of Traditional Chinese Medicine, Jinan, China

**Keywords:** mechanism, mitochondrial homeostasis, multi-organ protection, schisandrin B, translational challenges

## Abstract

Schisandrin B (Sch B) is a dibenzocyclooctadiene lignan derived from plants of the Schisandra genus. Owing to its pronounced lipophilicity, Sch B may readily cross biological membranes and is increasingly discussed in association with the regulation of mitochondrial homeostasis. Mitochondrial dysfunction underlies key pathological processes involved in multi-organ injury and a broad range of chronic diseases, manifested as redox imbalance, reduced mitochondrial membrane potential (ΔΨm), impaired ATP production, mitochondrial DNA (mtDNA) damage, disrupted mitochondrial dynamics, failure of mitochondrial quality control, and amplified inflammation, thereby promoting cell death and tissue remodeling. Accumulating evidence in recent years suggests that Sch B exerts biological effects associated with improved mitochondrial function in multiple models involving the liver, kidney, heart, brain, lung, and tumors. However, previous reviews have primarily focused on overall pharmacological activities or individual diseases, and a cross-organ integrative framework with “mitochondria” as the central axis remains limited. Based on current evidence, the mitochondria-related actions of Sch B can be summarized at several complementary levels: maintaining redox balance; stabilizing ΔΨm and potentially modulating the threshold of the mitochondrial permeability transition pore (mPTP); improving calcium homeostasis and bioenergetic output; reshaping the balance between fusion and fission; context-dependently regulating autophagy/mitophagy and autophagic flux; and bidirectionally influencing mitochondria-mediated apoptotic pathways in distinct cellular settings. At the organ level, the effects of Sch B exhibit a “pathology-driven matching” pattern: in acute stresses such as ischemia–reperfusion, Sch B tends to enhance mitochondrial stress tolerance and promote energy recovery; in toxin/drug-induced injury, it more prominently delays membrane structural disruption and oxidative damage; whereas in metabolic chronic diseases, its actions are associated with metabolic flexibility and the continuity of quality control processes. Despite the cross-organ consistency of Sch B in mitochondrial regulation, its translation remains constrained by factors including *in vivo* exposure, effective intramitochondrial concentration, delivery and targeting strategies, safety boundaries, and interindividual variability. Therefore, this review proposes a multi-organ mechanistic model centered on “mitochondrial homeostasis regulation,” providing a theoretical basis for understanding the cross-system effects of Sch B and for future drug development and optimization.

## 1 Introduction

**FIGURE 1:**
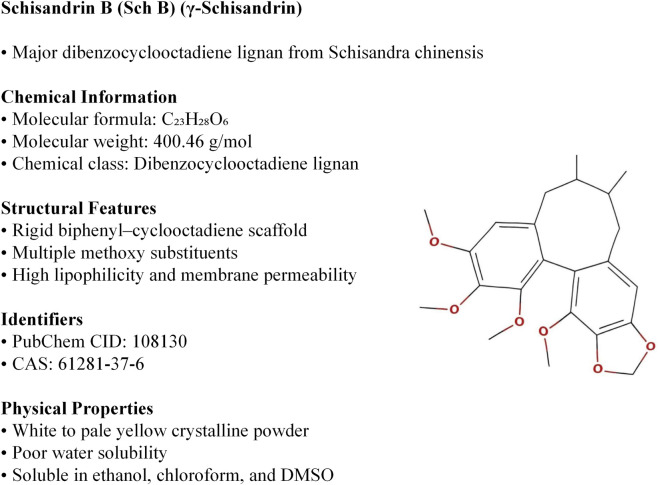
Chemical structure of Schisandrin B (Sch B), a representative dibenzocyclooctadiene lignan isolated from plants of the genus *Schisandra*. Major botanical sources include *Schisandra chinensis* (Turcz.) Baill. (Schisandraceae) and *Schisandra sphenanthera* Rehder & E.H. Wilson (Schisandraceae) (PubChem CID: 108130). Structural information was retrieved from the PubChem database, and the chemical structure was redrawn by the authors using WPS Office software for publication purposes. PubChem content is provided under public domain status, ensuring compliance with Frontiers attribution and licensing requirements.

Schisandrin B (Sch B) is a dibenzocyclooctadiene lignan isolated from plants of the genus *Schisandra*. The major botanical sources include *Schisandra chinensis* (Turcz.) Baill. (Schisandraceae) and *Schisandra sphenanthera* Rehder & E.H. Wilson (Schisandraceae). Schisandrin B (CAS 61281-37-6) has the molecular formula C_23_H_28_O_6_ (MW 400.46 g/mol) and contains multiple methoxy groups and stereogenic centers. It is a colorless to pale yellow crystalline solid, readily soluble in DMSO and alcohols but sparingly soluble in water (<0.1 mg/mL), and should be stored sealed and protected from light. Based on its dibenzocyclooctadiene scaffold with multiple aromatic rings and methoxy substituents, Sch B is generally considered a lipophilic lignan. Therefore, Sch B is expected to readily cross biological membranes (Kwan et al., 2017) (Figure 1).

*Schisandra chinensis* contains multiple structurally related dibenzocyclooctadiene lignans, such as schisandrin, schisandrin A, schisandrin C, and gomisins, many of which share partially overlapping antioxidant and cytoprotective activities and have been discussed in previous reviews. Compared with these related metabolites, Sch B has received particular interest because a growing number of studies have linked its pharmacological effects to mitochondrial functional regulation in different experimental contexts (Zhou et al., 2021). Importantly, the purpose of focusing on Sch B in this review is not to imply that it is the only active lignan in Schisandra, but rather that current evidence concerning Sch B is relatively enriched in mitochondria-associated mechanisms across multiple organ injury models. In addition, recent subcellular distribution studies have demonstrated that Sch B is detectable within cells and in isolated mitochondrial fractions (Meng et al., 2023), supporting the relevance of discussing its mitochondria-related actions.

Although several Schisandra lignans share a similar dibenzocyclooctadiene scaffold, their reported pharmacological emphases are not entirely identical. For example, schisandrin and schisandrin A have often been discussed in neuroprotective or anti-aging settings, whereas gomisin derivatives are frequently investigated in relation to hepatic metabolism and detoxification (Zhou et al., 2021). In comparison, Sch B has been increasingly examined for its potential involvement in mitochondrial functional regulation, including effects on mitochondrial membrane potential (ΔΨm), the mitochondrial permeability transition pore (mPTP), and mitochondrial redox balance in different organ injury models. Therefore, this review focuses on Sch B as a representative lignan with relatively abundant mechanistic evidence linking it to mitochondria-associated processes across multiple tissues.

Mitochondrial dysfunction is considered a common pathological basis of multisystem diseases, including liver injury, renal ischemia–reperfusion, myocardial ischemia, neurodegenerative diseases, osteoarthritis, and others. Mitochondrial damage can lead to excessive generation of reactive oxygen species (ROS), decreased mitochondrial membrane potential (ΔΨm), impaired ATP synthesis, mitochondrial DNA (mtDNA) damage, and inflammasome activation, ultimately triggering necrotic or apoptotic pathways. These pathological alterations span different tissue types, forming a high degree of unity in the “multi-organ injury–mitochondrial mechanism” axis (James and Murphy, 2002). Accordingly, mitochondria-centered intervention strategies have been considered a potential therapeutic direction across diseases and organs. In this context, Sch B represents a natural lignan that has been frequently investigated for its ability to modulate mitochondrial-related processes, which may contribute to organ-protective phenotypes under certain pathological conditions.

Importantly, the emphasis on Sch B in this review is not intended to exclude the pharmacological relevance of other Schisandra metabolites, but rather to highlight that Sch B provides a useful example for discussing mitochondria-associated protective mechanisms in a cross-organ context. Accumulating studies suggest that many of its reported effects tend to converge on mitochondrial homeostasis-related processes, which may offer a more coherent framework for interpreting its multi-organ protective and context-dependent actions than a purely general antioxidant description.

Current studies have demonstrated that Sch B can influence mitochondrial function through multiple pathways, including inhibiting ROS generation, activating the nuclear factor erythroid 2-related factor 2/antioxidant response element (Nrf2/ARE) antioxidant system, stabilizing ΔΨm, inhibiting opening of the mPTP, regulating the balance of mitochondrial dynamics, promoting autophagy/mitophagy, and suppressing mitochondria-mediated apoptotic signaling (Nasser et al., 2020). These mechanisms have been reported not only in the liver but also in the kidney, heart, nervous system, cartilage, and other tissues. Collectively, these findings suggest that Sch B may act on partially shared mitochondrial regulatory nodes across organs, while the dominant functional emphasis may vary depending on organ-specific vulnerability and pathological context.

In parallel, pharmacokinetic studies have shown that the plasma concentration–time curve after oral administration of Sch B presents a double-peak pattern, suggesting the presence of enterohepatic circulation; the absolute oral bioavailability in female rats is approximately 55%, which is significantly higher than that in males (approximately 19%), and there are sex differences in the area under the curve (AUC) and maximum plasma concentration (Cmax). Within the dose range of 10–40 mg/kg, the pharmacokinetics exhibit linear characteristics. The hepatic drug concentration is significantly higher than that in other tissues, followed by the kidney, suggesting that the kidney may participate in excretion; additionally, relatively high distribution is also observed in the ovary and adipose tissue, indicating that Sch B has extensive tissue distribution properties. The excretion of unchanged Sch B in urine, bile, and feces is extremely low, suggesting that Sch B is primarily eliminated through metabolic pathways, and metabolites constitute the major excreted forms rather than direct excretion of the parent compound (Zhu et al., 2013; Wang et al., 2018). These findings provide an important basis for formulation development, safety evaluation, and translational considerations, and they highlight the need to consider sex differences and enterohepatic circulation when interpreting pharmacological efficacy and exposure duration.

Although pharmacological studies on Sch B have continuously increased in recent years, existing reviews have often focused on its overall pharmacological activities or single disease models, while lacking a systematic summary of “mitochondria” as a critical functional axis. The commonalities and differences of mitochondrial mechanisms across different organs have not yet been comprehensively clarified, and an integrative analysis of multi-organ protective mechanisms of Sch B remains insufficient. Therefore, it is necessary to adopt “mitochondrial functional regulation” as the core perspective to systematically elucidate the roles of Sch B in various types of organ injury, thereby constructing a mechanistic model for its cross-organ effects and providing a theoretical basis for future drug development.

This review aims to focus on the regulatory actions of Sch B with mitochondria as a central functional axis across multiple organs, summarizing its major pharmacological processes in the liver, kidney, heart, brain, lung and tumor tissues, with particular emphasis on the shared mechanisms and organ-specific differences in mitochondrial redox regulation, dynamic balance, bioenergetic maintenance, autophagy modulation, and suppression of mitochondria-mediated apoptosis. On this basis, we propose a cross-organ model of Sch B as a potential mitochondria-centered protector, with the expectation of providing new directions for its preclinical research and subsequent development. It should be emphasized that the mitochondria-centered framing adopted in this review primarily reflects functional and mechanistic evidence, rather than definitive proof of direct mitochondrial targeting or enrichment. Quantitative data regarding intramitochondrial exposure, including free concentration, retention time, and dynamic distribution of Sch B, remain limited and represent a key translational gap.

## 2 Methods and search strategy

To clarify the mitochondria-associated pharmacological actions of Sch B (Sch B) across multiple organ injury and disease models, we conducted a structured literature search followed by a mechanistic narrative synthesis. This work was not intended to constitute a formal PRISMA-compliant systematic review or meta-analysis, but rather to provide an integrative cross-organ framework centered on mitochondrial homeostasis.

In accordance with the Four Pillars of Best Practice in Ethnopharmacology (pharmacological relevance, methodological quality, reproducibility, and taxonomic validity), particular attention was paid to the accurate identification of botanical sources, transparent evidence selection, and mechanistic interpretability of included studies. To ensure the accuracy of botanical source information, all plant names and related metabolites were taxonomically verified using the Medicinal Plant Names Services (MPNS; https://mpns.kew.org/mpns-portal/). In addition, the ConPhyMP tool (https://ga-online.org/best-practice/) was applied to support taxonomic consistency of plant materials and to meet best-practice requirements for ethnopharmacological research.

Comprehensive searches were performed in major databases including PubMed, Web of Science, and Google Scholar. The search primarily focused on experimental evidence describing the protective or regulatory effects of Sch B in organs such as the liver, kidney, heart, brain, lung, and tumor-related models, with emphasis on mitochondrial redox regulation, bioenergetic maintenance, mitochondrial dynamics, autophagy/mitophagy, and mitochondria-mediated apoptotic pathways. The publication time window was set from January 2010 to December 2025 to ensure methodological relevance and timeliness, while selectively citing earlier high-quality studies where appropriate. The final literature search was performed on 31 December 2025.

Search terms combined free-text keywords and controlled vocabulary when applicable. Core search strings included combinations of: “Schisandrin B” OR “Sch B” AND “mitochondria” OR “mitochondrial dysfunction,” together with terms such as “oxidative stress,” “mitophagy,” “mitochondrial permeability transition pore (mPTP),” “organ injury,” “ischemia–reperfusion,” “hepatotoxicity,” “nephrotoxicity,” “cardioprotection,” “neuroprotection,” “inflammation,” and “fibrosis.” As an example, the PubMed search included: (“Schisandrin B” [Title/Abstract] AND mitochondria [Title/Abstract]) combined with organ- or mechanism-specific terms.

Two independent reviewers (S.G. and F.Y.) screened titles, abstracts, and full texts according to predefined inclusion and exclusion criteria, with disagreements resolved through discussion with a third reviewer (Z.L.). Given the narrative and mechanistic focus of this review, no formal quantitative risk-of-bias scoring was applied; instead, heterogeneity across models, dosing regimens, and translational limitations are critically discussed throughout the manuscript.

## 3 Regulation of mitochondrial homeostasis by Sch B

### 3.1 Maintenance of mitochondrial redox homeostasis

Maintaining mitochondrial redox homeostasis has been proposed as an important process associated with the mitochondria-related effects of Sch B in a range of experimental settings. Under oxidative stress conditions, a reduced ratio of reduced glutathione/oxidized glutathione (GSH/GSSG) is a common feature of mitochondrial injury, and several studies have suggested that Sch B may influence the glutathione system as part of its antioxidant-related actions. Early work in healthy mice reported that Sch B increased mitochondrial GSH levels and decreased GSSG, which was interpreted as an improvement in the redox status of hepatic mitochondria (Ip et al., 1996). In chemical liver injury models, such as carbon tetrachloride (CCl_4_)-induced liver injury, Sch B was found to enhance mitochondrial glutathione antioxidant status (mtGAS) and to be associated with upregulation of heat shock protein 25/70 (HSP25/70). Notably, even when mtGAS enhancement was partially inhibited, hepatoprotective phenotypes were still observed, suggesting that antioxidant modulation may represent only one component of the broader mechanistic profile of Sch B (Chiu et al., 2003).

Evidence from other organ injury models has reported similar trends. In gentamicin-induced nephrotoxicity in rats, long-term Sch B administration was associated with improvements in renal injury indices, accompanied by increased mitochondrial GSH, α-tocopherol, and manganese superoxide dismutase (Mn-SOD) levels. These changes occurred alongside alterations in ATP-generating capacity, malondialdehyde (MDA) production, Ca^2+^ burden, and cytochrome c release, which were interpreted as reflecting mitigation of oxidative and mitochondrial stress (Chiu et al., 2008). In cerebral ischemia/reperfusion injury models, Sch B was likewise reported to increase mitochondrial glutathione and Mn-SOD levels in brain tissue and to reduce lipid peroxidation and calcium overload (Chen et al., 2008). In aging-related studies, long-term supplementation with Sch B was associated with changes in mitochondrial antioxidant indices and ATP-generating capacity across multiple tissues, although the extent to which these findings translate beyond specific animal models remains uncertain (Ko et al., 2008). Overall, while these preclinical studies collectively suggest a potential link between Sch B and mitochondrial redox regulation, the evidence is largely indirect and model-dependent, highlighting the need for further standardized and translational investigations.

At the molecular regulatory level, several studies have suggested that Sch B may influence redox-related signaling pathways, including the nuclear factor erythroid 2-related factor 2/Kelch-like ECH-associated protein 1 (Nrf2/Keap1) axis and its downstream antioxidant transcriptional network. Given that Nrf2 is a broadly responsive stress-regulatory factor activated by many phytochemicals, the specificity of Sch B–Nrf2 interactions remains an important point requiring further clarification. In an acute stress (forced swimming) model, Sch B was reported to promote Nrf2 nuclear translocation and to increase the levels of Nrf2-regulated antioxidant enzymes (such as SOD and enzymes related to GSH synthesis), accompanied by reduced ROS and MDA generation. These changes were interpreted as contributing to improved neuronal structural integrity under stress conditions (Wu et al., 2019). However, it should be noted that such outcomes are often influenced by model severity, dosing regimens, and the indirect nature of oxidative stress markers, which may limit cross-study comparability.

In an H9c2 hypoxia/reoxygenation model, (−)Sch B was found to induce glutathione production and antioxidant enzyme expression via the extracellular signal-regulated kinase/Nrf2 (ERK/Nrf2) pathway. Pharmacological inhibition of ERK or Nrf2 RNA interference attenuated these responses, and related findings were also reported in an *ex vivo* heart ischemia/reperfusion setting (Chiu et al., 2011). While these mechanistic interventions strengthen pathway plausibility, most evidence remains confined to simplified cellular systems, and whether comparable signaling hierarchies operate *in vivo* across organs has not been fully established.

In addition to antioxidant enzyme modulation, Sch B has been reported to be associated with reduced oxidative damage to mitochondrial DNA (mtDNA), induction of HSP25/70 expression, and preservation of mitochondrial oxidative phosphorylation (OXPHOS)-related function in certain experimental contexts. Nevertheless, mtDNA protection is often inferred from surrogate damage indices rather than direct quantification of mitochondrial genomic integrity or repair dynamics. In aging models, such effects were linked to attenuation of mtDNA damage and mitochondrial functional decline, although these observations remain largely confined to preclinical systems (Leong et al., 2012). Moreover, long-term supplementation paradigms may involve systemic metabolic adaptations beyond mitochondria-specific mechanisms, which should be considered when interpreting causality.

Taken together, available evidence suggests that Sch B may engage multiple layers of redox-related regulation, including modulation of the glutathione system, involvement of Nrf2-dependent antioxidant responses, and associations with mitochondrial structural preservation. At present, these pathways should be viewed as interconnected mechanistic hypotheses rather than definitive hierarchical cascades, because most studies rely on indirect functional readouts and vary substantially in experimental design. Therefore, while redox regulation represents a recurring theme in the literature, its role as a unifying or dominant mechanism of Sch B across tissues should be interpreted cautiously and warrants further standardized validation.

### 3.2 Stabilization of ΔΨm and inhibition of mPTP opening

Maintaining ΔΨm is generally considered an important prerequisite for preserving OXPHOS efficiency and limiting mitochondria-dependent apoptotic signaling, whereas ΔΨm decline and excessive mPTP opening frequently represent convergent events in stress-induced injury. Available evidence suggests that Sch B has been reported to influence mitochondrial functional parameters in diverse cellular and animal models, including stabilization of ΔΨm, modulation of mitochondrial Ca^2+^ homeostasis, altered sensitivity to permeability transition, and attenuation of cytochrome c release. However, it should be noted that ΔΨm and mPTP-related readouts are often assessed using indirect fluorescent probes, and differences in experimental conditions may affect cross-study comparability and mechanistic interpretation.

In a hypoxia/reoxygenation-induced hepatocyte injury model, Sch B (including (+/−)γ-Sch and (−)Sch B) increased GSH levels and improved ΔΨm, accompanied by decreased Ca^2+^-induced mitochondrial permeability transition sensitivity, reduced cytochrome c release, and attenuation of downstream cleavage of caspase-3 and PARP, which was interpreted as suppression of mitochondria-mediated apoptosis; notably, (−)Sch B exerted a more prominent protective effect (Chiu et al., 2009). While these findings support involvement of the Ca^2+^–mPTP node, the extent to which Sch B directly modulates permeability transition components *versus* acting through upstream antioxidant adaptation remains to be clarified.

In neuronal cell models, the effects of Sch B have also been linked to regulation of mitochondrial dynamics and bioenergetics. In H_2_O_2_-induced HT22 neuronal injury, Sch B was reported to maintain mitochondrial dynamic balance and stabilize ΔΨm by upregulating Sirt3 expression, resulting in an approximately 53% increase in cellular ATP production and preservation of mitochondrial number and structural integrity (Hu et al., 2025). Nevertheless, ATP restoration and dynamic remodeling may reflect broader metabolic stress responses, and causal hierarchy between ΔΨm stabilization and downstream survival outcomes requires further validation. This ΔΨm-centered regulation suggests that Sch B may contribute to cytoprotection through multiple mitochondrial-supportive processes rather than through a single isolated pathway.

In cardiac injury models, Sch B has frequently been discussed in relation to mPTP-associated regulation. In THP-induced cardiotoxicity in rats, Sch B increased SOD activity, reduced MDA levels, and was associated with inhibition of excessive mPTP opening, accompanied by reduced mitochondrial swelling and cytochrome c leakage, attenuation of cleaved caspase-9/3 activation, and changes in the Bcl-2/Bax ratio. These effects coincided with reduced cardiomyocyte apoptosis and improvement in functional and histological indices (Shi et al., 2021). In I/R injury models, Sch B was reported to inhibit mitochondrial Ca^2+^ overload, reduce ROS generation and cytochrome c release, enhance mitochondrial resistance to Ca^2+^-stimulated permeability transition, and attenuate cardiomyocyte apoptosis and tissue necrosis. Such findings suggest that modulation of the mPTP threshold may represent one plausible mechanism contributing to myocardial protection in these models (Chiu et al., 2007). At the same time, most supporting evidence remains preclinical, and direct target engagement within the mPTP complex has not been conclusively demonstrated, warranting cautious interpretation when discussing translational relevance.

Notably, the regulation of mPTP by Sch B appears to be context-dependent. In TGF-β1-induced activation models of HSC-T6 and LX-2 cells, Sch B instead upregulated Bax, downregulated Bcl-2, increased the Bax/Bcl-2 ratio, and induced mitochondrial permeability transition and cytochrome c release, thereby activating caspase-3 cleavage and promoting apoptosis (Li et al., 2021). This bidirectional behavior highlights that mitochondrial permeability regulation may vary substantially with cell type, activation status, and pathological context, rather than representing a uniformly protective mechanism. Such effects may be relevant for anti-fibrotic phenotypes in conditions characterized by abnormal proliferation.

In addition, in an LPS-induced mitochondrial injury model using C28I2 chondrocytes, Sch B was reported to maintain mitochondrial morphological stability by increasing fusion proteins and inhibiting fission protein expression, restore ΔΨm as indicated by JC-1 staining, and enhance mitochondrial activity measured by fluorescent probes, ultimately promoting cell proliferation and migration while reducing apoptosis (Hu et al., 2024). Because mitochondrial morphology is highly sensitive to inflammatory milieu and culture conditions, further studies using standardized quantitative approaches would be valuable to confirm the robustness of these observations.

In summary, current evidence suggests that Sch B may influence the ΔΨm–mPTP axis in multiple experimental systems, with reported effects on membrane potential stabilization, Ca^2+^ handling, energy metabolism, and apoptosis-related signaling. Conversely, in pathologically activated cells such as hepatic stellate cells, Sch B can induce apoptosis through permeability transition, underscoring its context-dependent bidirectional properties. Collectively, these findings support the ΔΨm–mPTP axis as a recurring mechanistic theme in the literature on Sch B, although its relative contribution across organs and its direct molecular specificity remain incompletely defined.

### 3.3 Regulation of mitochondrial dynamics

Mitochondrial dynamics (the balance between fusion and fission) provides an important structural basis for maintaining mitochondrial morphology, energy metabolism, and damage repair capacity. Dysregulated dynamics, particularly excessive fission, has been implicated in cellular injury processes in multiple tissues, including the liver, kidney, nervous system, and cartilage. Available studies suggest that Sch B may influence dynamic-related parameters from both structural and functional perspectives, including reported effects on fission/fusion-associated proteins and mitochondrial bioenergetic or antioxidant indices. However, because mitochondrial dynamics are highly responsive to cellular stress and metabolic state, distinguishing primary regulatory actions from secondary adaptive changes remains an important consideration.

Early studies in aging C57BL/6J mice suggested that Sch B may exert indirect regulatory effects on mitochondrial dynamics. Long-term supplementation with Sch B was associated with reduced age-related mitochondrial ROS generation, maintenance of antioxidant status, and preservation of energy metabolic efficiency across multiple tissues (brain, heart, liver, and kidney), accompanied by improvements in physiological decline (Ko et al., 2008). Given that oxidative stress and energetic deficiency are common upstream triggers of enhanced mitochondrial fission, these findings may reflect a permissive metabolic environment for dynamic balance rather than direct modulation of the fusion/fission machinery.

More recent evidence has explored potential mechanisms by which Sch B may regulate mitochondrial dynamics. In a hypoxia/reoxygenation model of HK-2 cells (mimicking renal ischemia–reperfusion) and in a mouse renal ischemia–reperfusion model, Sch B was reported to interact with AKT1 and promote its phosphorylation and mitochondrial localization, accompanied by altered expression of downstream dynamics-related effector proteins: DRP1 (a fission protein) was suppressed, whereas the fusion proteins OPA1 and MFN2 were increased (Xu et al., 2025). Although these findings provide a mechanistic hypothesis linking Sch B to AKT1-dependent signaling, the experimental basis for direct binding and the extent of target specificity warrant further validation using quantitative biochemical approaches. This regulation was associated with restoration of mitochondrial morphology toward a more network-like structure, alongside reduced ROS generation, improved ΔΨm and ATP production, and decreased apoptotic indices, coinciding with alleviation of renal ischemia–reperfusion injury. Nevertheless, because AKT signaling broadly influences cell survival pathways, it remains possible that mitochondrial dynamic changes represent one component of a wider cytoprotective response.

In neurodegenerative disease models, Sch B has also been reported to influence mitochondrial dynamics in conjunction with metabolic remodeling. In an amyloid-β1–42 (Aβ1–42) oligomer-induced primary hippocampal neuron Alzheimer’s disease (AD) model, Sch B restored ΔΨm, increased cytochrome c oxidase activity, reduced mPTP opening and cytochrome c release, and was accompanied by improvements in ATP production, citrate synthase activity, and glycolysis-related enzyme expression (Piao et al., 2021). More importantly, Sch B was reported to upregulate PGC-1α, enhance mitochondrial biogenesis, and partially restore the balance of fusion and fission proteins, thereby attenuating Aβ-associated mitochondrial fragmentation and energetic dysfunction. Because AD-related mitochondrial impairment involves complex and multifactorial pathways, further studies will be needed to determine whether these coordinated effects reflect direct regulation of dynamic machinery or broader improvements in neuronal metabolic resilience.

Taken together, current evidence suggests that Sch B may influence mitochondrial dynamic balance under multiple tissue contexts and injury conditions through interconnected mechanisms, including improvement of the redox environment, reported involvement of AKT1–DRP1/OPA1/MFN2-associated signaling, and concurrent changes in mitochondrial bioenergetics and biogenesis markers. At present, these findings should be interpreted within the limitations of predominantly preclinical models and indirect readouts of mitochondrial morphology. While modulation of injury-induced mitochondrial fragmentation represents a recurring observation, the relative contribution of dynamics regulation to organ-level outcomes remains to be further clarified in standardized and translationally relevant systems.

### 3.4 Promotion of mitophagy and restoration of autophagic flux

Mitophagy is generally regarded as an important mechanism for maintaining mitochondrial quality and cellular homeostasis through the selective clearance of damaged mitochondria. Available evidence suggests that Sch B may influence mitophagy-related processes, including recognition, sequestration, and degradation of mitochondria, potentially through autophagy-associated signaling axes such as AMPK/mTOR, TFEB, and PINK1/Parkin. However, it should be noted that mitophagy is often inferred from changes in autophagy markers or mitochondrial colocalization assays, and rigorous assessment of mitophagic flux remains technically challenging across different experimental systems. Depending on the pathological context, Sch B has been reported to either enhance or attenuate mitophagy-related activity, indicating a context-dependent regulatory pattern.

In fatty liver-related models, the autophagy-regulating effects of Sch B have been linked to mitochondrial quality control. In free fatty acid-stimulated HepG2 cells, mouse primary hepatocytes, and high-fat diet (HFD) mice, lipid droplet accumulation increases the burden of mitochondrial β-oxidation and contributes to mitochondrial stress. Under these conditions, Sch B was reported to activate the AMPK/mTOR inhibitory pathway, accompanied by enhanced autophagosome formation and restoration of autophagic flux. More importantly, Sch B was found to activate TFEB and an ATG5-dependent mechanism that not only promoted general autophagy but also enhanced lipid droplet clearance and fatty acid oxidation (Y et al., 2022). Because lipid metabolism, general autophagy, and mitophagy are tightly interconnected, it remains difficult to fully distinguish mitochondria-selective clearance from broader metabolic adaptation in these models. Nonetheless, mitophagy has been proposed as one component contributing to the removal of lipotoxicity-damaged mitochondria and partial restoration of oxidative capacity.

In ethanol-induced cardiac injury models, Sch B has been discussed in relation to regulation of “overactivated mitophagy.” Ethanol exposure upregulates NOX4 and causes excessive ROS accumulation, leading to mitochondrial damage. In response, cells activate autophagy and mitophagy as compensatory mechanisms. However, persistent ROS stimulation may drive mitophagy beyond physiological requirements, potentially contributing to mitochondrial depletion and apoptotic signaling. Sch B downregulated NOX4 and reduced ROS, accompanied by attenuation of abnormal mitophagy levels and reduced cleaved-caspase-dependent apoptosis (Tao et al., 2021). These findings suggest that Sch B may indirectly modulate mitophagy through upstream oxidative stress control, rather than acting as a dedicated mitophagy-specific regulator.

In the APAP-induced liver injury model, Sch B was reported to exert reparative effects on mitophagy-related clearance. Toxic metabolites produced during APAP metabolism impair mitochondrial membrane integrity, enhance oxidative stress, and suppress TFEB-mediated lysosomal function, resulting in blockade of mitophagic flux. Sch B inhibited EGFR and activated TFEB, upregulating autophagy markers such as Beclin-1 and LC3, while restoring autophagy–lysosome degradative capacity, thereby promoting clearance of damaged mitochondria (Li X. et al., 2023). In this context, the functional significance may lie less in increasing autophagy globally, and more in relieving lysosomal impairment that limits effective mitochondrial turnover.

In contrast, in a hepatic ischemia–reperfusion injury (HIRI) mouse model, early ischemia-triggered ROS surges induced excessive autophagy and mitophagy-associated stress responses. In this setting, Sch B downregulated Beclin-1 and LC3-II, accompanied by reduced activation of Bax and caspase-3/9, suggesting attenuation of autophagy–apoptosis coupling and mitochondria-mediated cell death (Fei et al., 2025). Because ischemia–reperfusion involves rapid and heterogeneous injury dynamics, the boundary between protective mitophagy and maladaptive overactivation may depend strongly on timing and severity, which should be considered when interpreting these findings.

In the Hepa1-6 hepatocellular carcinoma model, Sch B-induced autophagy has been linked to a “pro-death mitophagy” phenotype. Sch B-triggered ROS accumulation initiated autophagy and increased LC3 and Beclin-1 expression, alongside enhanced MDC-positive autophagosome formation. Tumor cells are often sensitive to mitochondrial stress, and ROS-associated mitophagy may contribute to ΔΨm reduction and activation of cell death pathways (Tan et al., 2022). Nevertheless, because autophagy in cancer can play dual roles depending on context, further mechanistic clarification is needed before assigning a definitive pro-death mitophagy interpretation.

Taken together, current evidence suggests that Sch B may modulate mitophagy-related pathways in a context-dependent manner, with reported enhancement of mitochondrial clearance in settings of accumulated damage (e.g., fatty liver or APAP-induced injury) and attenuation of excessive mitochondrial turnover in acute stress models such as ethanol exposure or ischemia–reperfusion. In tumor systems, Sch B-associated ROS stress may contribute to mitophagy-linked cell death phenotypes. At present, these observations collectively support mitophagy as a recurring mechanistic theme rather than a fully defined bidirectional “precision regulation” strategy, since most data remain preclinical and rely on indirect autophagy markers. Further studies incorporating standardized flux measurements, temporal resolution, and quantitative mitochondrial turnover assays will be essential to clarify the mechanistic specificity and translational relevance of Sch B in mitochondrial quality control.

### 3.5 Suppression of mitochondria-mediated apoptotic pathways

Mitochondria-mediated apoptosis is commonly driven by sequential events including ΔΨm loss, mPTP opening, cytochrome c (Cyt c) release, and caspase-9 activation, and represents a major execution mechanism of cell death under stress, metabolic dysregulation, and certain antitumor settings. Available evidence suggests that the effects of Sch B on this pathway may be highly context-dependent: in several tumor cell models, Sch B has been reported to enhance intrinsic apoptosis through disruption of mitochondrial homeostasis, whereas in non-malignant tissues such as the stressed heart, it has more often been associated with attenuation of excessive apoptosis through stabilization of mitochondrial function (Xu et al., 2011). However, because apoptotic signaling is influenced by cell type, baseline metabolic vulnerability, and experimental dosing conditions, the apparent bidirectionality should be interpreted cautiously rather than as a universally defined regulatory rule.

In glioma models, Sch B-associated apoptosis has been linked to mitochondrial injury. Following Sch B treatment in U87/U251 cells, ΔΨm decreased, accompanied by Bax upregulation and Bcl-2 downregulation, shifting the Bax/Bcl-2 ratio toward a pro-apoptotic profile; subsequently, Cyt c release and caspase-9/caspase-3 activation were observed, leading to PARP cleavage and increased apoptotic indices (Li et al., 2015). Tumor growth inhibition in xenograft settings was consistent with these molecular events, suggesting that mitochondrial apoptotic signaling may contribute to the anti-glioma phenotype. Nevertheless, xenograft outcomes are shaped by multiple systemic factors, and further work is needed to clarify the extent to which mitochondrial apoptosis represents the dominant driver of tumor suppression *in vivo*.

In hepatocellular carcinoma and breast tumor cells, Sch B has been reported to enhance DOX-triggered mitochondrial apoptosis. Co-treatment exacerbated ΔΨm loss, increased Cyt c release, and strengthened activation of caspase-9 (rather than caspase-8), suggesting preferential engagement of the intrinsic pathway (Li et al., 2006). Notably, the same treatment did not induce a comparable increase in apoptosis in primary cardiomyocytes or fibroblasts, implying some degree of tumor-selective vulnerability. However, such selectivity remains model-dependent and may reflect differences in baseline oxidative stress sensitivity, proliferative state, or drug exposure between malignant and non-malignant cells, rather than an inherently tumor-specific targeting mechanism.

In triple-negative breast tumor (TNBC), Sch B has been linked to disruption of mitochondrial homeostasis through inhibition of STAT3-associated survival signaling. Upon STAT3 inhibition, TNBC cells exhibited Bax upregulation and Bcl-2 downregulation, accompanied by decreased ΔΨm, Cyt c release, and activation of caspase-9/caspase-3, while reductions in tumor volume *in vivo* were also reported (Dai et al., 2018). These findings suggest that, despite differences in upstream triggers, apoptotic execution under Sch B treatment frequently converges on mitochondrial pathways. At the same time, STAT3 signaling regulates broad cellular programs beyond mitochondria, and mechanistic specificity should be further validated through direct mitochondrial target engagement studies.

In gallbladder tumor models, Sch B similarly induced mitochondria-associated apoptosis, including decreased ΔΨm (monitored by JC-1), increased Bax/Bcl-2 ratio, and activation of caspase-9/caspase-3 with PARP cleavage; inhibition of NF-κB activity further weakened anti-apoptotic capacity (Xiang et al., 2014). Because these studies largely rely on canonical apoptotic markers, additional investigation is required to determine whether Sch B initiates apoptosis primarily through mitochondrial disruption or through coordinated multi-pathway stress responses.

In contrast to tumor contexts, an opposite regulatory direction has been reported in myocardial I/R injury models. Under I/R conditions, cardiomyocytes display ΔΨm loss, Cyt c release, and activation of caspase-9/caspase-3, accompanied by ER stress signaling such as ATF6, PERK, and CHOP. Sch B treatment was associated with improved ΔΨm stability, reduced Cyt c release, and an increased Bcl-2/Bax ratio, coinciding with inhibition of mitochondria-mediated apoptosis (Zhang et al., 2017). Meanwhile, Sch B suppressed the ATF6/PERK–CHOP axis, suggesting attenuation of ER–mitochondrial stress coupling. These protective observations highlight the possibility that Sch B may act as a stress-modulating agent in non-malignant tissues, although the therapeutic window and translational applicability require further study.

Taken together, Sch B has been reported to influence mitochondria-mediated apoptosis through changes in ΔΨm stability, Bax/Bcl-2 balance, Cyt c release, and caspase execution cascades, with directionality varying across pathological contexts. Rather than indicating a strict upstream–downstream consistency, current evidence supports a context-sensitive association between Sch B and mitochondrial apoptotic signaling that depends on cell type, injury state, and experimental conditions. This context dependence may partially explain its reported dual relevance in antitumor models and organ injury settings, while also underscoring the need for cautious interpretation and further mechanistic clarification.

**FIGURE 2:**
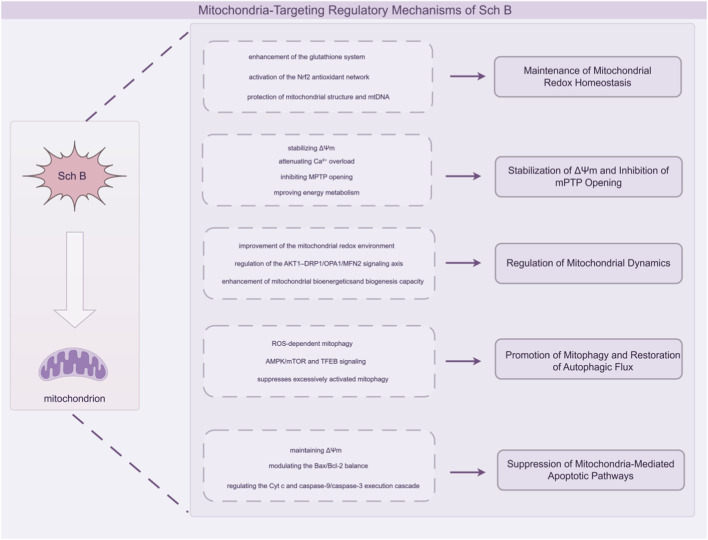
This figure illustrates the integrated mechanisms by which Sch B exerts mitochondrial protective effects. Sch B enhances the glutathione system, activates the Nrf2 antioxidant network, and preserves mitochondrial structure and mtDNA, thereby maintaining mitochondrial redox homeostasis. It stabilizes the mitochondrial membrane potential (ΔΨm), reduces Ca^2+^ overload, inhibits mPTP opening, and improves mitochondrial bioenergetic function. In addition, Sch B modulates the AKT1–DRP1–OPA1/MFN2 signaling axis to maintain mitochondrial dynamic balance and enhance bioenergetics and biogenesis. It also regulates ROS-dependent mitophagy via the AMPK/mTOR and TFEB pathways, restoring autophagic flux and preventing excessive mitochondrial clearance. Ultimately, Sch B suppresses mitochondria-mediated apoptosis by maintaining ΔΨm, regulating the Bax/Bcl-2 ratio, and inhibiting cytochrome c release and caspase-9/caspase-3 activation, highlighting its coordinated regulation of mitochondrial homeostasis.

Overall, Sch B has been reported to influence mitochondrial function through multi-level mechanisms that converge on mitochondrial homeostasis, although most supporting evidence remains preclinical and model-dependent. Studies suggest that Sch B may modulate redox-related parameters via the glutathione system and Nrf2-associated signaling, accompanied by reduced oxidative damage indices and supportive effects on mitochondrial structural maintenance. In parallel, Sch B has been associated with stabilization of ΔΨm, altered sensitivity to Ca^2+^-induced mPTP opening, and changes in ATP-generating capacity in multiple experimental systems, although these functional readouts are often indirect and context-sensitive. Sch B has also been linked to regulation of fusion/fission-related proteins, including reported involvement of the AKT1–DRP1/OPA1/MFN2 axis, as well as context-dependent effects on autophagy/mitophagy activity. Importantly, the direction of these effects appears to vary across pathological settings, and mitophagy-related conclusions frequently rely on marker-based inference rather than standardized flux measurements. At the level of apoptotic execution, Sch B has been reported to enhance intrinsic apoptosis in several tumor cell models, while attenuating excessive apoptosis in non-malignant tissue injury settings such as myocardial ischemia. Taken together, current findings suggest that the mitochondria-associated actions of Sch B are highly context-dependent, and further studies incorporating quantitative target engagement and translational validation are required before definitive mechanistic hierarchies can be established (Figure 2).

## 4 Organ-targeted actions of Sch B

### 4.1 Liver: mitochondrial protection under metabolic stress

Among multiple organs, the liver is often considered highly dependent on mitochondrial homeostasis because of its central metabolic functions. As the metabolic hub of the body, the liver undertakes fatty acid β-oxidation, the urea cycle, gluconeogenesis, and detoxification, and therefore sustains substantial OXPHOS flux to meet energetic demands. However, this metabolic specialization may also render hepatic tissue particularly sensitive to mitochondrial imbalance in drug-induced liver injury (DILI), alcoholic liver disease (ALD), non-alcoholic fatty liver disease (NAFLD), and acute toxic injury models such as APAP and CCl_4_. Under these injury contexts, hepatic mitochondria frequently exhibit early pathological alterations, including impaired OXPHOS, excessive ROS generation, decreased ΔΨm, oxidative damage to mtDNA, and disruption of autophagic flux (Ramachandran et al., 2021; Gao and Bataller, 2011; Prasun et al., 2021). These mitochondrial dysfunctions are closely associated with hepatocyte apoptosis or necrosis, and may amplify inflammatory cascades through mtROS-mediated activation of the NLRP3 inflammasome, while also participating in TGF-β1-driven activation of hepatic stellate cells (HSCs). Such processes are regarded as contributing mechanisms in the progression from early liver injury toward fibrosis (Wu X. et al., 2017). Nevertheless, the relative contribution of each mitochondrial pathway may vary across disease etiologies and experimental models, highlighting the heterogeneity of hepatic mitochondrial stress responses.

More importantly, mitochondria support the metabolic flexibility of hepatocytes across physiological and pathological states, enabling dynamic adjustment of energy flux and redox balance during fasting, nutrient excess, oxidative stress, or toxic exposure. However, this reliance on structure–function coupling also means that the liver is vulnerable to mitochondrial homeostasis disruption, and recurring mitochondrial pathological patterns can be observed across injury paradigms. Therefore, small molecules capable of modulating mitochondrial-associated nodes have been proposed as potential intervention candidates in liver disease, although translational applicability requires careful evaluation of exposure, safety boundaries, and disease-stage specificity.

In APAP-induced liver injury, formation of N-acetyl-p-benzoquinone imine (NAPQI) rapidly depletes hepatocellular glutathione, weakening antioxidant defenses and disrupting mitochondrial electron transport, leading to an acute energy crisis. Under these conditions, mitochondrial uncoupling is aggravated and ATP levels decline rapidly, compromising ionic homeostasis and membrane integrity and resulting in extensive hepatocellular necrosis (Jaeschke et al., 2012). In this context, the actions of Sch B have been discussed as involving integrated stress-modulating capacity rather than a single defined target. Studies have reported that in APAP models, Sch B enhances hepatic mitochondrial tolerance to acute metabolic insults, including activation of metabolic pathways (such as the pentose phosphate pathway) to maintain antioxidant substance levels, and inhibition of signaling pathways (such as MAPK–JNK–ERK) associated with apoptosis and inflammation (Cheng et al., 2022). These findings suggest a buffering effect against acute metabolic stress, although the degree of protection is likely model-dependent and influenced by dosing and timing relative to injury onset.

Distinct from the APAP model, CCl_4_-induced hepatotoxicity emphasizes membrane structural disruption driven by free-radical chain reactions. Following generation of trichloromethyl radicals, polyunsaturated fatty acids in mitochondrial membranes are preferentially attacked, increasing lipid peroxidation and affecting mitochondrial morphology, cristae stability, and the microenvironment of electron transport chain proteins (Fareed et al., 2024). Under these conditions, studies have observed that Sch B is associated with enhanced tolerance to lipid peroxidation, including increased membrane antioxidant capacity and reduced oxidative modification of membrane proteins, thereby delaying structural deterioration under oxidative stress (Zhang et al., 2019). These findings are consistent with a membrane-stabilizing phenotype. A plausible explanation is that the lipophilicity of Sch B may facilitate membrane partitioning, but direct evidence for inner-mitochondrial-membrane association or quantitative intramitochondrial exposure remains limited. Thus, mechanistic interpretation should remain cautious, and membrane-related effects may represent one component within broader antioxidant adaptation.

In lipotoxic and metabolic liver disease models such as NAFLD/non-alcoholic steatohepatitis (NASH), mitochondrial injury is more often driven by chronic metabolic overload rather than acute toxic stimulation. Impaired fatty acid oxidation, reduced mitochondrial density, decreased electron transport efficiency, and weakened OXPHOS capacity are considered major features (Sunny et al., 2017). Long-term lipid accumulation may disturb mitochondrial architecture and dynamic balance, contributing to reduced fusion and increased fission. Evidence suggests that in NAFLD models, Sch B has been associated with “metabolic re-coordination”, including improved handling of fatty acid load, maintenance of functional mitochondrial density, and enhancement of antioxidant systems (Leong and Ko, 2016). However, these effects may be strongly influenced by dosage, intervention window, and the chronicity of metabolic stress, limiting direct extrapolation across disease stages.

ALD models further reflect hepatic mitochondrial sensitivity to toxin metabolism byproducts. Acetaldehyde and ethanol-induced ROS place mitochondrial proteins and lipids under persistent oxidative modification, increasing protein-folding burden and impairing respiratory chain efficiency and β-oxidation (Teschke, 2018). Meanwhile, ALR, a hepatoprotective factor, is downregulated after alcohol intake, contributing to reduced antioxidant capacity and impaired ΔΨm (Verma et al., 2022). Given the cumulative nature of mitochondrial injury in ALD, mitochondrial renewal and replacement efficiency are considered relevant for slowing progression. In this context, studies have reported that Sch B may promote mitochondrial biogenesis-related processes and enhance mitophagy, allowing partial replacement of damaged mitochondria under long-term exposure (Tao et al., 2021). Notably, such effects appear less prominent in short-term injury paradigms, suggesting disease-stage dependence of mitochondrial quality-control modulation.

In inflammation and fibrosis models, mitochondrial injury displays cell type-specific features. Mitochondrial dysfunction in hepatocytes primarily affects energy metabolism and survival, whereas mitochondrial status in Kupffer cells is linked to inflammatory cytokine release. The metabolic state of HSCs influences their transition from quiescent to activated phenotypes and represents an important basis for fibrogenesis (Ezhilarasan, 2021). Studies have shown that in fibrosis-related models, Sch B exerts metabolic regulatory effects in HSCs, reducing oxidative metabolic intensity and suppressing mitochondrial respiratory capacity, thereby weakening activation-associated energy supply and fibrogenic potential (Li et al., 2021). This cell type-dependent behavior further indicates that mitochondrial regulation by Sch B in the liver is multidimensional rather than uniformly protective across all hepatic populations.

It should also be noted that some studies have raised concerns regarding potential cytotoxicity of Sch B in liver-related cells. In mouse hepatocyte (AML-12) and macrophage (RAW 264.7) models, Sch B under specific conditions has been reported to induce cytotoxic responses, including cell cycle arrest, alteration of Bcl-2 family balance with caspase activation, and autophagy-related changes through inhibition of PI3K/Akt/mTOR signaling (Zhang et al., 2015). These findings suggest that Sch B may exert pro-apoptotic effects at higher doses or in susceptible contexts, emphasizing that dose dependence, safety margins, and interindividual variability should be carefully considered in translational evaluation.

### 4.2 Kidney: hypoxia vulnerability and mitochondrial protection

As a key organ responsible for maintaining fluid and electrolyte homeostasis, the kidney—particularly proximal tubular epithelial cells—exhibits a metabolic phenotype that is highly dependent on OXPHOS. Renal cells possess relatively high mitochondrial density and undertake energy-intensive tasks of ion transport and active reabsorption. Unlike the metabolic specialization of the liver, the anatomical structure of the kidney determines that it operates under a pronounced corticomedullary oxygen tension gradient. This unique microenvironment places the kidney in a long-term metabolic state characterized by the coexistence of “high energy demand and relatively low oxygen supply”. Accordingly, renal mitochondrial homeostasis displays marked oxygen sensitivity: when microcirculatory obstruction, increased metabolic load, or toxin exposure disrupts this balance, renal mitochondria may shift toward dysfunction, manifested by reduced energy supply, elevated ROS levels, mtDNA damage, and disturbed dynamics (Zhang et al., 2023; Zhang et al., 2021; Che et al., 2014). Nevertheless, the extent of mitochondrial vulnerability and downstream injury outcomes may vary substantially across nephron segments and disease contexts, reflecting the heterogeneity of renal metabolic adaptation. These features suggest that mitochondria-associated modulation has been proposed as a potential intervention direction in kidney injury, although translational relevance requires careful validation.

In I/R injury, this metabolic vulnerability is often further amplified. During ischemia, interruption of oxygen supply rapidly suppresses OXPHOS, resulting in ATP depletion; upon reperfusion, sudden restoration of oxygen can trigger ROS surges, ΔΨm disturbance, and mitochondrial morphological changes, thereby promoting inflammatory signaling and cellular injury (Huang et al., 2024; Li H. et al., 2025). This type of injury involves multidimensional mitochondrial imbalance driven by abrupt oxygen fluctuations rather than a single pathway abnormality. Under such intense metabolic insult, available evidence suggests that Sch B does not converge on a single molecular target but has been associated with enhancement of mitochondrial stress tolerance in RIRI models. Studies have reported that Sch B attenuates ROS peaks and supports structural stability and energy recovery dynamics during ischemia–reperfusion (Xu et al., 2024). However, because ΔΨm and ROS readouts are often probe-dependent and timing-sensitive, further standardized studies are needed to clarify the robustness and mechanistic specificity of these observations. In addition, regulation of mitochondrial dynamics by Sch B has been proposed to contribute to functional stability during phases of rapid remodeling (Piao et al., 2021). Overall, the potential relevance of Sch B in I/R settings may lie in buffering mitochondrial responses to oxygen tension shifts, although such effects remain largely preclinical.

In drug-induced nephrotoxicity models, renal mitochondrial vulnerability arises through different mechanisms. Relative accumulation of toxins in the kidney may lead to intracellular exposure that damages membrane proteins, lipids, and mtDNA, driving progressive mitochondrial injury (Lash, 2021; Gai et al., 2020). Direct intramitochondrial accumulation, however, is often difficult to quantify, and mechanistic conclusions should be interpreted cautiously without exposure-based measurements. Unlike I/R injury, stress in these models derives from sustained chemical stimulation and gradual deterioration of membrane integrity. Under this context, Sch B has been associated with suppression of lipid peroxidation, delayed cristae disruption, and partial preservation of ΔΨm and Ca^2+^ homeostasis (Liu et al., 2011). For example, in nephrotoxicity models induced by aminoglycosides, heavy metals, or cyclosporine, studies have reported that Sch B supports mitochondrial antioxidant capacity and basal metabolic function (Chiu et al., 2008; Lai et al., 2017). Given its lipophilicity, Sch B may partition into biological membranes, potentially contributing to membrane-associated effects, although direct evidence for mitochondrial membrane enrichment remains limited. Thus, in drug-induced renal injury, Sch B has been discussed as a modulator that may delay structural deterioration rather than fully preventing toxicity progression.

Chronic injury models, such as DKD and lipotoxic renal injury, represent another trajectory of mitochondrial damage. Their features involve long-term hyperglycemia, hyperlipidemia, and inflammatory microenvironment-driven metabolic reprogramming, resulting in gradual declines in mitochondrial efficiency, increased fragmentation, and enhanced stress status (Cle et al., 2023; Ahmad et al., 2021). Podocytes, owing to their specialized structure, are particularly sensitive to energy imbalance and may develop filtration barrier instability under sustained mitochondrial exhaustion. Under such chronic overload, Sch B has been reported to influence mitochondrial biogenesis markers, dynamic balance, oxidative metabolic efficiency, and extracellular matrix (ECM) deposition, thereby supporting coordinated energy output in experimental models (Liu et al., 2023). However, chronic metabolic diseases involve multifactorial systemic remodeling, and the relative contribution of mitochondrial regulation *versus* broader metabolic effects remains to be clarified.

It is noteworthy that different renal cell types show marked differences in metabolic demand and mitochondrial dependence: proximal tubular cells are vulnerable to acute energy crisis, podocytes are sensitive to chronic metabolic stress, whereas medullary cells adapt to hypoxic environments (Duann and Lin, 2017). The differential emphasis of Sch B across models may reflect these subtype-specific metabolic constraints. Overall, current evidence suggests that Sch B may exert context-dependent mitochondria-associated effects in renal injury paradigms, including stress buffering in ischemia–reperfusion, membrane-related stabilization in nephrotoxicity, and metabolic support in chronic disease models. At present, these findings support an emerging framework rather than definitive proof of broad renal protection, and further studies incorporating quantitative mitochondrial exposure, target engagement, and translational endpoints are needed before Sch B can be positioned as a candidate for renal mitochondrial intervention.

### 4.3 Heart: mitochondrial protection under high energy demand

The heart relies on continuous mechanical pumping activity with extremely high energy demand, and more than 90% of ATP is derived from mitochondrial OXPHOS, making mitochondria a key structural and functional component for maintaining excitation–contraction coupling in cardiomyocytes. Unlike the liver and kidney, cardiomyocyte mitochondria are densely distributed and embedded within the myofibrillar structure in a highly organized manner, enabling tight coupling between energy production and mechanical output. Although this structure–function integration maximizes cardiac efficiency, it also renders the heart sensitive to stimuli that disrupt mitochondrial homeostasis. In models of I/R injury, heart failure (HF), hypertrophic stress, chemotherapy-induced cardiotoxicity, and metabolic cardiomyopathy, mitochondrial dysfunction has been consistently implicated in disease progression and contributes to cardiac metabolic vulnerability (Bisaccia et al., 2021). Nevertheless, the relative importance of specific mitochondrial pathways may vary across disease etiologies, stages, and experimental models, underscoring the heterogeneity of cardiac mitochondrial injury.

In I/R injury, ischemia causes electron transport chain stagnation and accumulation of metabolic intermediates, whereas abrupt restoration of oxygen supply during reperfusion triggers ROS bursts, Ca^2+^ overload, and mitochondrial structural disruption, promoting rapid energetic collapse and cell injury (Pedriali et al., 2022). Under this acute metabolic insult, Sch B has been reported to enhance tolerance of cardiac mitochondria to early reperfusion-associated fluctuations, including suppression of oxidative stress, attenuation of ER stress-related apoptosis, stabilization of ΔΨm, and reduced Ca^2+^-triggered permeability transition. These effects were accompanied by improvements in infarct-related and functional indices in experimental models (Chiu et al., 2007; Zhang et al., 2017; Zhao et al., 2018). However, most evidence remains confined to animal and *ex vivo* systems, and the magnitude of benefit may depend strongly on dosing, timing of administration, and injury severity. In permanent ischemia models (e.g., LAD ligation), Sch B was also associated with reduced remodeling-related markers, including altered eNOS activity and inflammatory responses, suggesting broader metabolic support under sustained ischemia (Chen et al., 2013). Such findings suggest a buffering role in acute energy crisis, although mechanistic hierarchy remains incompletely defined.

In pressure overload and HF models, cardiac metabolic stress reflects a persistent mismatch between energy demand and supply. With sustained pressure load (e.g., TAC), cardiomyocytes undergo hypertrophy, metabolic reprogramming, and gradual decline in mitochondrial function, including reduced fatty acid oxidation, cristae disruption, ROS elevation, and imbalanced dynamics (Lopaschuk et al., 2021; Shao et al., 2022; Wei et al., 2024). Under this chronic stress, Sch B has been reported to attenuate MAPK-associated hypertrophy and fibrosis, reduce oxidative burden, and preserve mitochondrial functional output, coinciding with improvements in remodeling indices (Ai et al., 2019). Nevertheless, chronic HF involves complex systemic neurohormonal and inflammatory remodeling, and the extent to which mitochondrial modulation alone accounts for functional outcomes remains to be clarified. Thus, Sch B may contribute to maintenance of metabolic flexibility in these settings, consistent with broader observations in chronic metabolic disease models.

In metabolic diseases such as diabetic cardiomyopathy (DCM), chronic hyperglycemia and hyperlipidemia induce impaired substrate utilization and lipid peroxidation, resulting in “chronic energy deficiency with aggravated oxidative injury”. Declines in ATP production, ΔΨm impairment, and insufficient antioxidant capacity contribute to progression. Studies have reported that Sch B modulates myocardial lipid metabolism, enhances antioxidant indices, and improves ΔΨm and ATP-related parameters, accompanied by functional improvements in experimental DCM models (Niu et al., 2025). However, because metabolic cardiomyopathy develops over long timeframes, the translational relevance of such interventions will require evaluation in clinically representative models and exposure windows.

In drug-induced cardiotoxicity models (e.g., doxorubicin, cisplatin, and trastuzumab), mitochondrial injury mechanisms show drug specificity: doxorubicin inhibits respiratory complexes and promotes iron-mediated oxidative injury; cisplatin induces mtDNA damage and ΔΨm loss; trastuzumab involves ErbB2 signaling disruption and altered membrane permeability (Wallace et al., 2020; Oliveira et al., 2024; Ye et al., 2023). Despite distinct upstream triggers, these injuries converge on mitochondrial dysfunction and energetic insufficiency. Sch B has been reported to exert multi-parameter effects in these models, including suppression of oxidative stress, stabilization of ΔΨm, reduced abnormal mPTP opening, improved ATP generation, and attenuation of apoptotic signaling, coinciding with reductions in myocardial enzyme release and structural injury markers (Shi et al., 2021; Tang et al., 2022). At present, these observations support potential mitochondrial stress-modulating properties, although direct clinical extrapolation remains premature without systematic safety and pharmacokinetic validation.

It is also noteworthy that different cardiac cellular subtypes exhibit differences in mitochondrial dependence. Cardiomyocytes rely on oxidative metabolism for contractility; fibroblasts, endothelial cells, and immune cells contribute to fibrosis, vascular homeostasis, and inflammation through mitochondrial signaling (Yang, 2025). Although current studies have not systematically characterized Sch B actions across these cell types, the recurring mitochondrial-related trends suggest that its effects may not be restricted to a single cellular compartment. Future work incorporating cell type-resolved approaches will be important to clarify tissue-level integration.

From an integrated perspective, Sch B has been associated with context-dependent mitochondrial modulation in cardiac disease models: buffering acute energetic collapse in I/R, supporting metabolic adaptation in pressure overload and HF, improving substrate handling in metabolic cardiomyopathy, and attenuating mitochondrial degeneration under drug toxicity. These findings collectively support an emerging mechanistic framework rather than definitive proof of broad cardioprotection. Future studies incorporating high-resolution mitochondrial imaging, single-cell metabolomics, and stress kinetics analyses will be valuable for defining regulatory patterns and evaluating translational relevance.

### 4.4 Nervous system: mitochondrial vulnerability and neuroprotection

The nervous system is regarded as one of the organ systems most sensitive to mitochondrial homeostasis, owing to its extreme dependence on energy supply, highly refined axonal microstructural organization, and limited regenerative capacity. Even at rest, neurons must sustain substantial basal metabolism to support maintenance of ionic gradients, synaptic transmission, and axonal transport, with approximately 90% of energy derived from mitochondrial OXPHOS. Meanwhile, neuronal mitochondria display strong regional specialization: presynaptic terminals require high energy to sustain vesicle cycling; long axonal projection regions depend on mitochondrial trafficking and stationary anchoring; and dendritic spines involve mitochondria in local protein synthesis and synaptic plasticity. Therefore, any pathological event that interferes with mitochondrial structure, transport, density distribution, or metabolic flexibility may trigger structural and network-level amplification effects in the nervous system, manifested as synaptic failure, axonal degeneration, and even neuronal death. Accordingly, mitochondrial dysfunction is considered a common early driving factor in neurodegenerative diseases, ischemic brain injury, inflammatory neuropathies, and chemotherapy-induced neurotoxicity (Misgeld and Schwarz, 2017; Alshial et al., 2023; Bustamante-Barrientos et al., 2023). However, it should be emphasized that mitochondrial dysfunction in these diseases is often multifactorial, and it remains unclear whether mitochondrial impairment is the primary cause or a secondary consequence of broader cellular dysfunction.

In cerebral ischemia and reperfusion injury (CIRI) models, mitochondrial damage often precedes morphological cellular changes. During ischemia, inhibition of the electron transport chain and interruption of ATP synthesis lead to abrupt declines in energy production and accumulation of metabolic intermediates; during reperfusion, rapid restoration of oxygen supply triggers massive ROS generation and disrupted calcium homeostasis, resulting in mitochondrial swelling, cristae disruption, and network fragmentation, accompanied by imbalanced fusion/fission dynamics (Huang et al., 2023). Such a “failed restart” mitochondrial state represents an important bioenergetic node determining differential cell fate between the infarct core and the penumbra. While these findings underscore the importance of mitochondrial dysfunction in determining cell survival during reperfusion, the lack of direct quantification of mitochondrial dynamics and energy flux during these transitions limits our understanding of the precise mechanisms. In this model, studies have shown that Sch B enhances antioxidant capacity in brain mitochondria (e.g., increasing glutathione, α-tocopherol, and Mn-SOD levels and reducing lipid peroxidation) and alleviates structural disruption, thereby improving mitochondrial function during early reperfusion and promoting overall brain tissue survival (Chen et al., 2008). These changes primarily reflect an integrated improvement of mitochondrial antioxidant status and structural integrity, enabling mitochondria to maintain a certain level of respiratory chain activity during reperfusion fluctuations, rather than a simple “free radical scavenging” effect. It is important to note that while antioxidant effects are crucial, other factors such as mitochondrial membrane stability and the regulation of mitochondrial dynamics may also play significant roles in determining the overall protection. In cerebral I/R models, Sch B has also been shown to reduce inflammatory cytokine levels and matrix metalloproteinase activity after focal cerebral ischemia, thereby alleviating tissue injury and edema (Lee et al., 2012). Across these ischemic models, the core action of Sch B can be interpreted as enhancing the “mitochondrial tolerance threshold” of neurons to reperfusion stress by improving oxidative stress resistance and structural stability, thereby reducing the transition from energy imbalance to irreversible cell death, rather than relying on transient suppression of a single signaling pathway. However, the clinical relevance of these preclinical findings remains uncertain, and further studies using translational models are needed to determine whether Sch B could provide similar neuroprotection in human patients.

In neurodegenerative diseases such as AD, PD, and HD, mitochondrial injury manifests as long-term and persistent energy decline and failure of quality control. Reduced respiratory chain activity, insufficient ATP production, mtDNA damage, impaired mitochondrial trafficking, disruption of fusion/fission balance, and decreased mitochondrial density in synaptic regions are common features across multiple neurodegenerative pathologies. Meanwhile, protein aggregation (e.g., Aβ, tau, and α-synuclein) directly affects mitochondrial membrane structure and respiratory efficiency, exacerbates ROS and oxidative stress, and forms a vicious cycle of sustained metabolic deterioration. Under these chronic pathological contexts, Sch B has shown supportive effects on long-term mitochondrial homeostasis in multiple models, including enhancing mitochondrial antioxidant reserves, maintaining ΔΨm stability, delaying cristae degeneration, promoting mitochondrial renewal, and optimizing mitochondrial distribution within neurons (Monzio Compagnoni et al., 2020; Klemmensen et al., 2024). In chronic cerebral hypoperfusion models, Sch B improves axonal mitochondrial transport patterns, enabling synaptic regions to receive relatively stable energy supply under long-term energy stress (He et al., 2024); in PD and AD models, Sch B reduces mitochondrial oxidative burden and interference by protein aggregation, ultimately reflected by a slower rate of mitochondrial capacity decline (Ba et al., 2015; Giridharan et al., 2015). While Sch B has shown beneficial effects in improving mitochondrial function in neurodegenerative models, it is critical to note that these effects are context-dependent and may vary depending on disease stage and the underlying pathology. These effects can be summarized as follows: Sch B does not directly reverse degenerative pathology, but sustains neuronal metabolic flexibility under chronic stress, allowing longer preservation of synaptic function and structural integrity. However, translating these effects into clinical applications remains uncertain, as these models are primarily preclinical and may not fully replicate the complexity of human neurodegenerative diseases.

In inflammatory neural injury, mitochondria serve as both a damage target and an amplifier of inflammatory signaling. ROS and cytokines released by inflammatory cells can damage neuronal mitochondrial membrane lipids and reduce respiratory chain efficiency; conversely, mitochondrial oxidative pressure and mtDNA leakage can further drive amplification of inflammatory cascades, forming a bidirectional “inflammation–mitochondrial injury” loop (Willemen et al., 2023). Under this bidirectional injury environment, the mitochondrial protective effects of Sch B in neurons include reducing oxidative burden, enhancing antioxidant capacity, maintaining mitochondrial membrane stability, and limiting structural collapse during energy supply restriction, thereby delaying neuronal apoptosis and synaptic loss under high inflammatory stress (Jiang et al., 2015). In parallel, Sch B reduces mitochondria-driven inflammatory output in microglia, thereby lowering the external stress load imposed on neuronal mitochondria and helping to prevent further spread of inflammation (Yang Y. et al., 2024). However, further studies are needed to distinguish between direct mitochondrial effects and broader immunomodulatory actions, as inflammation is a complex and multifactorial process involving various immune cell types.

In chemical neuroinjury and peripheral neuropathy models, multiple chemotherapeutic agents directly act on mitochondria by inhibiting the electron transport chain, damaging mtDNA, or disrupting Ca^2+^ regulation, thereby inducing energy crisis and axonal degeneration (Shen et al., 2023). Available *in vitro* studies suggest that Sch B slows mitochondrial energy system collapse under high oxidative stress conditions, including maintaining basal ATP output, stabilizing ΔΨm, enhancing redox buffering capacity, and preserving mitochondrial network morphology (Hu et al., 2025; Lam and Ko, 2012; Leong et al., 2014). These findings suggest that Sch B may provide protective effects in certain models of chemotherapy-induced neurotoxicity, but its clinical applicability remains uncertain and warrants further validation. Together, these changes indicate that Sch B can maintain the minimal functional threshold of mitochondria under sustained chemical stress, thereby preventing neurons from rapidly entering irreversible injury.

It should be noted that mitochondrial dependence varies markedly among neural cell types. Excitatory neurons, due to extremely high energy demand and predominant reliance on OXPHOS to sustain continuous firing and synaptic activity, are highly sensitive to mitochondrial functional decline. Inhibitory neurons similarly require local mitochondria to provide rapid and stable energy support for synaptic inhibitory precision and network rhythm maintenance. In contrast, glial cells (especially astrocytes) exhibit greater metabolic flexibility; their mitochondrial status not only determines their capacity to provide metabolic substrates to neurons but also affects efficiency in maintaining redox homeostasis and local supportive functions (Bélanger et al., 2011). Although mitochondria-specific actions of Sch B across these cellular subtypes have not been fully elucidated, existing trends suggest that Sch B may enhance energy stability in neurons while reducing mitochondria-derived stress in glial cells, thereby conferring integrated protection at the network level. Further cell-type specific studies are needed to fully define the differential actions of Sch B in these cell populations.

Overall, the nervous system, characterized by highly structured energy demand, precise regional mitochondrial distribution, and amplification of minor perturbations, places mitochondria at the core of its pathological vulnerability. Current evidence indicates that Sch B confers systemic neuroprotection across models of acute ischemia, chronic degeneration, inflammatory injury, and chemical injury by increasing mitochondrial stress tolerance thresholds, maintaining structural and functional stability, improving energy output, and reducing mitochondrial-related signaling imbalance. However, further research incorporating mitochondrial trafficking imaging, dynamic measurements of synaptic energy flux, and brain organoid models will be essential to better elucidate the mitochondrial regulatory potential of Sch B and its clinical cardioprotective value.

### 4.5 Lung: mitochondrial homeostasis under high oxygen pressure

As an organ continuously exposed to a high-oxygen environment, the lung exhibits mitochondrial biological features that are markedly distinct from those of other tissues. Alveolar epithelial cells, alveolar macrophages, and vascular endothelial cells must maintain stable OXPHOS under hyperoxic conditions to support gas exchange, surfactant synthesis, and immune surveillance. However, long-term high oxygen tension in the alveolar region also means that the mitochondria of these cells operate close to a physiological boundary characterized by “high oxidative pressure with limited buffering reserve”, making them more prone to mitochondrial overload and crossing the homeostatic threshold during infection, inflammation, mechanical ventilation, or environmental toxin exposure (Schumacker et al., 2014). Therefore, mitochondrial vulnerability in the lung is not simply “more ROS”, but rather reflects that once redox buffering capacity is further compressed, energy supply, membrane structural stability, and immune homeostasis can become synchronously imbalanced and amplified through cascading processes. It should also be noted that lung mitochondrial responses are highly cell type- and context-dependent, and hyperoxia-related stress mechanisms may differ substantially between experimental models and human disease settings.

The role of mitochondria in ALI exhibits a clear “energy–oxidation–structure” coupling pattern. First, reduced OXPHOS efficiency results in insufficient ATP supply, preventing alveolar epithelial and endothelial cells from maintaining tight junctions and ionic homeostasis under stress. Second, impaired electron transport chain function rapidly elevates mtROS, driving lipid peroxidation and accelerating ΔΨm instability and cristae disruption. Third, damaged mitochondria release mtDNA and other DAMPs, amplifying innate immune responses and forming a positive feedback loop of “mitochondrial injury–inflammation amplification–further mitochondrial injury” (Zhan and Shen, 2022). While this conceptual framework is supported by multiple studies, the relative contribution of each mitochondrial component may vary depending on injury triggers, pathogen burden, and the timing of inflammatory escalation. In infection-induced ALI, this loop is more readily pushed toward dysregulation: pathogen-related stimulation and the hyperoxic inflammatory environment accelerate mitochondrial functional failure in lung cells and disrupt metabolic adaptation of immune cells, making inflammatory responses more difficult to discriminate and terminate effectively (Cloonan and Choi, 2016). Meanwhile, collapse of mitochondrial energy supply in epithelial and endothelial cells directly weakens the alveolar–capillary barrier, leading to aggravated leakage, pulmonary edema, and diffusion impairment (McClintock et al., 2022).

Under this acute “threshold collapse” background, multiple LPS-induced ALI studies have shown that Schisandrin-related components and Sch B can significantly alleviate lung pathological injury. However, it is important to distinguish between Sch B-specific effects and broader lignan mixture effects, as well as to recognize that most evidence remains based on acute endotoxin-driven models rather than clinically heterogeneous ALI/ARDS settings. Within a mitochondrial framework, the effects of Sch B can be more specifically described at three mutually supportive levels. First, Sch B reduces oxidative damage burden during the peak of acute inflammation, limiting mtROS-driven chain lipid peroxidation reactions and thereby slowing the decline of inner mitochondrial membrane structure and ΔΨm (reflected by reduced oxidative damage and cell death at the tissue level). Second, Sch B indirectly extends the “energy-sustaining window” of alveolar epithelial and endothelial cells, reducing the probability of rapid barrier disruption caused by ATP insufficiency, which is consistent with improvements in endpoints such as decreased wet-to-dry ratio and alleviated pulmonary edema. Third, Sch B suppresses the tendency of “mitochondrial danger signal release–inflammation re-amplification” within the injury amplification loop, making damaged mitochondria less likely to accumulate and drive secondary injury (reflected by reduced tissue destruction accompanied by decreased inflammatory cell infiltration and inflammatory mediators) (Cai et al., 2016; Zhu et al., 2023). Nevertheless, many of these conclusions are inferred from histological and biochemical markers rather than direct mitochondrial respiration or flux-based measurements, highlighting the need for further mechanistic precision. These mechanisms do not require attributing the effects to a single signaling axis, but are more consistent with a protective pattern of “increasing mitochondrial stress tolerance threshold under combined hyperoxia and inflammation, and delaying synchronous collapse of energy and structure”.

When disease progresses into a chronic stage (e.g., allergic asthma or smoking-related inflammation), lung mitochondrial injury is more characterized by “long-term deterioration” rather than “instantaneous collapse”. Persistent oxidative pressure gradually reduces the effective mitochondrial pool, decreases respiratory efficiency, and progressively induces network fragmentation and insufficient renewal, causing epithelial cells to shift toward low-metabolism and low-repair states, while inflammation is more likely to persist under metabolically inefficient conditions. In disease models, on the one hand, Sch B reduces oxidative stress and lipid peroxidation burden under long-term exposure, preventing continuous worsening of mitochondrial membrane systems and cristae structure, thereby slowing the decline in the proportion of functional mitochondria. On the other hand, Sch B enhances intracellular reductive buffering capacity and reduces accumulation of mitochondrial damage induced by chronic stress, enabling epithelial cells to retain a degree of metabolic flexibility and repair potential (reflected by improved histopathology and reduced inflammatory burden) (Chen Y. et al., 2021; Chen X. et al., 2021; Jia et al., 2017). However, chronic airway diseases involve complex immune remodeling and structural adaptation, and whether mitochondrial modulation alone can meaningfully alter long-term clinical trajectories remains uncertain. Therefore, in chronic lung injury, Sch B appears to shift mitochondria from a “continuously declining trajectory” back toward a “maintainable homeostatic range,” with its value more reflected in delaying disease progression and preserving metabolic resilience rather than reversing structural changes in a single step.

In pulmonary fibrosis models, mitochondrial homeostasis disruption becomes further consolidated as a driver of structural remodeling. Decreased mitochondrial oxidative capacity and accumulated oxidative injury in epithelial cells weaken effective post-injury repair and push tissues toward persistent stress and abnormal repair states, thereby providing a background for collagen deposition (Schumacker et al., 2014). In the BLM-induced pulmonary fibrosis model, Sch B reduces collagen deposition and improves oxidative stress-related indicators. Mechanistically, this phenomenon can be further refined. First, Sch B limits cumulative attacks of long-term oxidative injury on mitochondrial membrane lipids and mtDNA, reducing the probability of “damaged mitochondria continuously producing mtROS and danger signals”, thereby alleviating persistent stress and senescence tendencies in epithelial cells (Wang et al., 2020). Second, Sch B reduces accumulation of functionally impaired mitochondria by promoting turnover and clearance of damaged components, enabling cells to regain a certain degree of energy and redox balance capacity, thereby weakening the metabolic basis of “repair failure–abnormal remodeling” during fibrotic progression (Yang J. et al., 2024). Nonetheless, fibrosis progression is driven by multiple stromal and immune interactions, and mitochondrial-centered regulation should be viewed as one upstream contributor rather than a singular determinant. Thus, in PF, Sch B is more likely to act on the upstream process of “mitochondrial deterioration and loss of repair capacity” rather than directly targeting collagen deposition as the terminal phenotype.

PH models further illustrate, from a vascular perspective, the relationship between pulmonary mitochondrial homeostasis and phenotypic transition. Hypoxia-induced pulmonary arterial smooth muscle cells often undergo metabolic reprogramming, with suppressed OXPHOS and a stronger proliferative and migratory advantage, which constitutes an important basis for vascular remodeling (Schumacker et al., 2014). In hypoxia-induced PASMC models, Sch B has been reported to inhibit abnormal proliferation and improve remodeling-related phenotypes. Sch B may reduce the “proliferative advantage” jointly driven by metabolic inefficiency and oxidative pressure by limiting further decline in mitochondrial function under hypoxia, thereby functionally weakening the metabolic support required for vascular remodeling (Wu J. et al., 2017). However, it should be noted that direct measurements of mitochondrial respiration, ΔΨm, or mtROS sources in PASMCs remain relatively insufficient; therefore, this part should still be regarded as a reasonable synthesis based on metabolic pathological features rather than a fully experimentally confirmed conclusion. Future work incorporating quantitative bioenergetic assays will be essential to strengthen mechanistic interpretation in vascular pulmonary contexts.

In summary, continuous hyperoxic exposure and the high dependence of barrier function on energy supply make the lung particularly prone to mitochondrial homeostasis disruption that triggers multi-center yet convergent pathological chains. In the acute phase, mitochondrial energy collapse and structural disruption lead to rapid barrier failure; in the chronic phase, accumulated mitochondrial deterioration results in reduced repair capacity and structural remodeling; and vascular models reflect metabolic reprogramming-driven abnormal proliferation and remodeling. Available evidence supports a clear “pathology-aligned” pattern of Sch B across different lung injury models. At present, these findings should be interpreted as an emerging preclinical framework rather than definitive proof of clinical pulmonary protection. This mitochondria-centered regulatory pattern, which provides cross-model explanatory power, positions Sch B as a potential candidate compound warranting further systematic validation in key pulmonary cell subsets using mitochondrial functional assays and high-resolution imaging.

### 4.6 Tumor system: mitochondrial adaptability and therapeutic intervention

The tumor system exhibits the most complex and dynamic mitochondrial metabolic features among systemic diseases. Its core characteristic is not a generalized decline in mitochondrial function, but rather a marked enhancement of mitochondrial adaptability and metabolic reprogramming capacity. Under multiple selective pressures, including rapid proliferation, hypoxia and nutrient limitation, immune stress, and therapeutic interventions, tumor cells often restructure mitochondrial function to obtain a “survival buffer.” On the one hand, they maintain or even reinforce OXPHOS to support ATP supply and reducing equivalent generation; on the other hand, they redirect the tricarboxylic acid cycle and its branches to provide biosynthetic precursors, while sustaining homeostatic output in extreme microenvironments through plasticity of mitochondrial membrane structure and network dynamics. Meanwhile, oncogene- and tumor suppressor-driven remodeling of metabolic networks, accumulation of oncometabolites caused by mutations in metabolic enzymes, and the central roles of mitochondria in setting cell death thresholds, metabolic dependence of tumor stem cells, and metabolic competition within the tumor microenvironment collectively position mitochondria as key regulatory nodes for tumor progression and treatment response (Liu and Shi, 2020; Chen et al., 2024). For this reason, tumor mitochondria serve not only as a major source of adaptive advantage, but also as an exploitable vulnerability in therapeutic intervention: once mitochondrial “adaptive reserve” is continuously compressed or homeostasis is disrupted, tumor tolerance to hypoxia, oxidative pressure, and pharmacological stress will be markedly reduced. However, mitochondrial plasticity varies substantially across tumor types and metabolic phenotypes, and the extent to which mitochondrial vulnerability can be therapeutically exploited remains context-dependent.

Within this framework of “coexisting mitochondrial adaptability and vulnerability”, evidence across multiple tumor models indicates that the antitumor effects of Sch B can be unified as compression of the adjustable homeostatic space of tumor cell mitochondria, thereby increasing the probability of crossing the death threshold. At present, most supporting evidence derives from *in vitro* systems and xenograft models, and whether these mitochondrial threshold effects translate into clinically meaningful tumor control remains to be established. In colorectal tumor cells and xenograft models, Sch B-induced cellular stress is not confined to a single stress pathway, but displays a clear mitochondrial coupling to ultimate cell fate: as stress levels rise, tumor cells more readily enter irreversible apoptotic programs, and tumor growth *in vivo* is significantly suppressed (Co et al., 2024a). These findings suggest that Sch B increases tumor sensitivity to endogenous stress, making it difficult to maintain mitochondria-related survival homeostasis, thereby transforming metabolic adaptability that normally supports tumor survival into a therapeutic vulnerability. Nevertheless, the degree of stress sensitization may depend on dosage, exposure duration, and tumor metabolic reliance on OXPHOS *versus* glycolytic pathways.

In nasopharyngeal carcinoma models, the enhancement of radiosensitivity by Sch B can likewise be incorporated into the logic of mitochondrial threshold regulation. Studies have shown that Sch B selectively inhibits proliferation of nasopharyngeal carcinoma cells and induces cell cycle arrest, while weakening post–DNA damage repair capacity, thereby amplifying the cytotoxic effects of radiotherapy (Fang et al., 2025). Radiotherapy imposes pronounced oxidative pressure and energetic burden; when cell cycle progression is restricted and damage repair is impaired, tumor cells become less able to traverse the “high ROS–high energy demand” danger window through metabolic reprogramming and mitochondrial homeostatic compensation, and are therefore more likely to trigger mitochondria-dependent death outcomes. Thus, the radiosensitizing effect of Sch B in this model reflects compression of the compensatory space through which tumor cells maintain mitochondrial homeostasis under radiotherapy stress. However, radiosensitization remains highly dependent on tumor oxygenation status and microenvironmental heterogeneity, which are difficult to fully recapitulate in experimental systems.

More direct evidence comes from multiple digestive system tumor models. Studies in gallbladder tumor and cholangiocarcinoma consistently report that Sch B treatment induces ΔΨm decline and activates intrinsic apoptotic cascades, accompanied by cell cycle arrest and suppression of tumor growth *in vivo*. In these models, ΔΨm reduction is not only a hallmark event of apoptosis, but also reflects compromised mitochondrial bioenergetic output and membrane structural integrity, enabling tumor cells to more readily cross the mitochondrial death threshold and enter irreversible apoptosis (Xiang et al., 2014; Yang et al., 2016). These studies present “ΔΨm impairment–apoptotic cascade initiation–tumor growth suppression” as a relatively closed evidence chain, suggesting that the antitumor action of Sch B is closer to a systemic weakening of tumor mitochondrial adaptability. Nonetheless, ΔΨm changes can represent both cause and consequence of apoptotic execution, and further work is needed to establish whether mitochondrial perturbation constitutes a primary initiating event or a downstream amplification step.

In gastric tumor models, the antitumor effects of Sch B can also be integrated within a framework of “altered mitochondrial stress threshold”. On the one hand, studies report that Sch B inhibits tumor cell proliferation and affects autophagy-related processes, thereby enhancing synergistic cytotoxicity with chemotherapeutic agents such as 5-FU (He et al., 2022). On the other hand, Sch B has been reported to induce oxidative stress and associate with cell cycle arrest and apoptotic outcomes, and these effects can be partially reversed by antioxidant intervention (Li T. et al., 2023). Taken together, Sch B in gastric tumor cells may either increase oxidative burden to approach the upper limit of mitochondrial tolerance, driving membrane functional imbalance and apoptotic outcomes, or alter the handling of damaged organelles, reshaping mitochondrial quality control and stress outcomes. However, autophagy modulation in tumors can exhibit dual pro-survival or pro-death roles, and mechanistic interpretation requires careful context-specific evaluation. This “ROS pressure–mitochondrial threshold–cell fate” structure is consistent with the biological positioning of mitochondria as integrators of tumor stress.

In the context of treatment resistance, Sch B further appears to weaken mitochondria-related survival advantages. In DOX-resistant breast tumor and ovarian tumor models, Sch B reduces drug efflux and increases effective intracellular drug exposure, while also attenuating anti-apoptotic advantages, thereby enhancing sensitivity to death signals and reversing resistant phenotypes (Wang et al., 2017). Resistant cells commonly rely on a higher “death threshold” and a more robust mitochondrial survival buffer to counter chemotherapy-induced oxidative pressure and energy crises. By simultaneously weakening both the limitation on drug impact intensity and the mechanisms elevating the death threshold, Sch B effectively compresses the mitochondrial homeostatic window from both the input side and the threshold side, making resistant cells less able to maintain mitochondrial function and more likely to enter irreversible death outcomes (Nasser et al., 2019). Nevertheless, reversal of resistance in experimental models does not necessarily predict durable responses in heterogeneous clinical tumors, where compensatory pathways may emerge. In prostate tumor models, Sch B-induced oxidative stress accompanied by cell cycle arrest and apoptosis further suggests that Sch B may inhibit tumor proliferation by increasing mitochondria-related stress burden and compressing compensatory space (Lv et al., 2015).

In lung adenocarcinoma A549 and glioma models, Sch B similarly exhibits a combined effect of “cell cycle suppression–mitochondria-mediated apoptosis initiation–reduction of invasion-related phenotypes,” and suppresses tumor growth in xenograft models. ΔΨm decline, Cyt c release, and caspase cascade activation indicate that the effects of Sch B are not limited to slowing tumor proliferation, but rather enhance the triggerability of mitochondrial death pathways, shifting tumor cells from a state of “high adaptive survival” toward a metabolic edge with “low maintainable homeostasis” (Li et al., 2015; Murphy and Hartley, 2018). However, xenograft outcomes are influenced by host immune absence and simplified microenvironmental structure, requiring cautious interpretation when considering translational applicability.

A key challenge in mitochondrial regulation within tumor systems lies in selectivity: mitochondrial adaptability must be weakened in tumor cells while avoiding equivalent disruption of mitochondrial homeostasis in normal cells. In hepatocellular carcinoma and breast tumor models, Sch B enhances doxorubicin-induced mitochondrial apoptotic features in tumor cells while exhibiting differential responses in normal cardiomyocytes and fibroblasts (Li et al., 2006). This phenomenon suggests the presence of a metabolic context-dependent selectivity window, but it remains insufficient to support clinical-level inferences that Sch B can reduce cardiotoxicity or broadly decrease chemotherapy dosage. More systematic *in vivo* safety and pharmacokinetic validation remains required. In particular, quantitative assessment of mitochondrial exposure and target engagement in tumor *versus* normal tissues will be essential for defining such selectivity.

**FIGURE 3:**
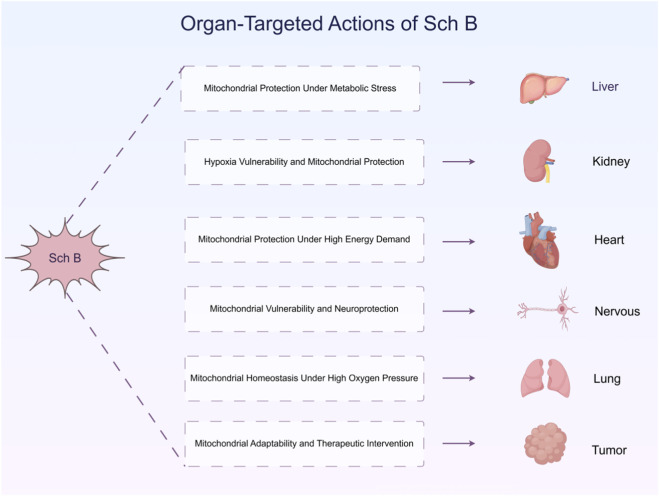
This figure summarizes the organ-targeted actions of Sch B through its regulation of mitochondrial function under diverse pathological conditions. Sch B confers mitochondrial protection in the liver during metabolic stress, enhances mitochondrial resilience in the kidney under hypoxic conditions, and supports mitochondrial function in the heart under high energy demand. In the nervous system, Sch B mitigates mitochondrial vulnerability and contributes to neuroprotection, while in the lung it maintains mitochondrial homeostasis under high oxygen pressure. Additionally, Sch B improves mitochondrial adaptability in tumor tissues, highlighting its potential therapeutic relevance. Overall, the figure emphasizes that Sch B exerts broad organ-specific protective effects by modulating mitochondrial stability, adaptability, and stress responses across multiple tissues.

Overall, mitochondria in the tumor system are characterized by high plasticity, dynamic behavior, and strategic adjustment. Tumor cells expand mitochondrial adaptive reserve to resist stress and acquire therapeutic tolerance, whereas the common directionality of Sch B across multiple tumor models is compression of this mitochondrial adaptive reserve: by increasing oxidative burden to approach mitochondrial tolerance limits, disrupting ΔΨm and energy homeostasis to enhance the triggerability of death pathways, or weakening resistance-associated threshold-elevation mechanisms to potentiate therapeutic cytotoxicity. At present, these findings support an emerging mechanistic synthesis rather than definitive evidence of broad antitumor efficacy, and the contribution of mitochondrial regulation relative to other stress pathways remains to be clarified. Future studies should further define the mitochondrial selectivity window of Sch B across tumors with different metabolic phenotypes and, by integrating metabolomics, mitochondrial functional assays, tumor organoids, and single-cell analyses of the tumor microenvironment, systematically evaluate its translational value from an integrated perspective of mitochondrial structure–function–threshold (Figure 3).

This table summarizes representative *in vivo* and *in vitro* studies investigating Sch B in models of acute and chronic organ injury, inflammatory diseases, neurodegeneration, and tumor systems. For each study, experimental model, Sch B dosing regimen (including route and timing), principal mitochondria-related mechanisms (e.g., redox homeostasis, ΔΨm stabilization, mPTP regulation, mitochondrial dynamics, autophagy/mitophagy modulation, and mitochondria-dependent apoptosis), and major functional outcomes are standardized for cross-study comparison. Dosing units are presented as reported in the original studies to avoid conversion-related bias. Abbreviations are used consistently throughout the manuscript. To reduce the risk of overly naive reporting of pharmacological activities, evidence summarized in Table 1 was extracted with attention to key parameters required for pharmacological relevance and methodological interpretability. Specifically, we recorded the experimental model type (*in vitro* vs. *in vivo*), dosing or concentration range, timing of intervention (pretreatment/co-treatment/treatment), and the presence of mechanistic or pharmacological controls where reported (e.g., pathway inhibitors or positive reference compounds).

**TABLE 1:** Representative evidence supporting Sch B–mediated mitochondrial regulation across organ injury and disease models.

References	Experimental type + subjects	Drug and dose	Mechanisms	Outcomes
Ip et al. (1996)	*In vivo*: female Balb/c mice, CCl_4_-induced acute liver injury	Sch B 1201 mg/kg/day, gavage, pretreatment once daily for 3 days before CCl_4_ administration	Mitochondrial glutathione system: mtGSH↑, mtGSSG↓ → mtGSH/GSSG ratio↑ (not directly caused by increased GRD activity)	Nearly completely blocked acute liver injury under CCl_4_-induced oxidative stress
Chiu et al. (2003)	*In vivo*: female Balb/c mice, CCl_4_-induced acute liver injury	Sch B 801 mg/kg, gavage, single pretreatment 24 h before CCl_4_ administration	mtGSH↑; mtGRD↑, mtGPX↑, mtGST↑; HSP25↑/HSP70↑; mitochondrial energetics: ATP-generating capacity preserved; mitochondrial lipid peroxidation reduced (mtMDA↓)	Significantly alleviated CCl_4_-induced liver injury; maximal protection observed at 24 h after dosing
Chiu et al. (2009)	*In vitro*: AML12 mouse hepatocytes, H/R (2 h + 16 h)-induced apoptosis	(±)γ-Sch or (−)Sch B 2.5–5.0 μM, pretreatment for 24 h before hypoxia/reoxygenation	Cellular antioxidant defense: GSH↑; mPTP opening↓; ΔΨm↑; mitochondrial apoptosis: Cyt c release↓, caspase-3/PARP cleavage↓	Markedly inhibited H/R-induced apoptosis and improved cell viability
Li et al. (2021)	*In vitro*: rat HSC-T6 and human LX-2 hepatic stellate cells, TGF-β1 (10 ng/mL)-induced activation	Sch B 1.56–25 μM, co-treatment for 24 h with TGF-β1	Inhibition of activation: α-SMA↓, Collagen I↓, TIMP1↓; mitochondria-related apoptosis: Bax↑, Bcl-2↓, cleaved caspase-3↑	Inhibited hepatic stellate cell proliferation and promoted apoptosis, thereby blocking profibrotic phenotypes
Fei et al. (2025)	*In vivo*: male C57BL/6J mice, hepatic I/R (ischemia 90 min + reperfusion 24 h)	Sch B 30 mg/kg/day, gavage, pretreatment once daily for 7 days before hepatic I/R	Autophagy-related: Beclin-1↓, LC3-II↓; apoptosis-related: Bax↓, caspase-3/9↓; rapamycin partially reversed the effects	Significantly attenuated hepatic I/R injury and reduced inflammation and tissue necrosis
Tan et al. (2022)	*In vitro*: mouse hepatoma Hepa1-6 cells	Sch B 25, 50, and 100 μM, treatment for 24 h	ROS↑ → autophagy activation↑; selenoproteins↓ (Txnrd1/2/3, GPX1/2, etc.), associated with autophagy and immune-factor alterations; antioxidant indices decreased (GSH/SOD/GSH-Px↓)	Inhibited hepatoma cell proliferation and induced prominent autophagic phenotypes, accompanied by Th1-skewed immune factor expression
Li et al. (2015)	*In vivo*: subcutaneous U87 xenografts in nude mice *In vitro*: U87/U251 glioma cells	*In vitro*: Sch B 25, 50, and 100 μM, treatment for 48 h; *In vivo*: Sch B 25 or 100 mg/kg, intraperitoneal injection, administered every 2 days for 20 days	Mitochondrial apoptosis: ΔΨm↓; Bcl-2/Bax ratio↓; cleaved caspase-9/3↑, PARP cleavage↑; cell cycle: Cyclin D1/CDK4↓ (G0/G1 arrest)	Strongly inhibited glioma cell proliferation and induced apoptosis; markedly suppressed tumor growth *in vivo*
Li et al. (2006)	*In vitro*: SMMC7721 hepatocellular carcinoma cells and MCF-7 breast cancer cells; normal controls: primary rat cardiomyocytes/human dermal fibroblasts	Sch B 50 μM combined with DOX 1 μM, co-treatment for 48 h	Enhanced mitochondrial apoptosis: caspase-9 activation↑ (not caspase-8); ΔΨm loss↑ → caspase-3↑, PARP cleavage↑; independent of P-gp/MRP1, DOX uptake, or ROS	Enhanced DOX-induced apoptosis in cancer cells, with no evident sensitizing effect in normal cells
Dai et al. (2018)	*In vivo*: MDA-MB-231 xenografts in nude mice *In vitro*: TNBC cells (MDA-MB-231/BT-549/468)	*In vitro*: Sch B 50–100 μM, treatment for 24 h; *In vivo*: Sch B 50 or 100 mg/kg/day, intraperitoneal injection, treatment once daily	STAT3 pathway: p-STAT3 Tyr705↓, nuclear translocation↓; blocked IL-6–induced STAT3 activation; downstream: Bcl-2/Bcl-xl↓, cleaved caspase-3↑; cell cycle arrest (G0/G1 or G2/M)	Suppressed TNBC cell proliferation and migration and induced apoptosis; significantly inhibited tumor growth *in vivo* without obvious organ toxicity
Xiang et al. (2014)	*In vivo*: NOZ xenografts in nude mice *In vitro*: GBC-SD/NOZ gallbladder cancer cells	*In vitro*: Sch B 30, 60, and 90 μM, treatment for 48 h; *In vivo*: Sch B 30 or 100 mg/kg, intraperitoneal injection, administered every 2 days for 15 days	Mitochondrial apoptosis: ΔΨm↓; Bax↑, Bcl-2↓; cleaved caspase-9/3↑, PARP cleavage↑; NF-κB↓; cell cycle: Cyclin D1/CDK4↓ (G0/G1 arrest)	Inhibited gallbladder cancer cell proliferation and induced apoptosis; markedly suppressed xenograft growth *in vivo*
Zhang et al. (2017)	*In vivo*: male SD rats, myocardial I/R (LAD 40 min + reperfusion 1 h)	Sch B 20/40/80 mg/kg, gavage, pretreatment once daily for 5 days before I/R	ER stress: PERK/p-PERK↓, ATF6↓, CHOP↓; apoptosis: Bax↓, caspase-9/3↓, Bcl-2↑; oxidative stress: MDA↓, T-SOD↑	Significantly attenuated myocardial I/R injury and improved histological and functional outcomes
Cheng et al. (2022)	*In vitro*: HHL-5 human embryonic liver cells, APAP (20 mM)-induced injury	Sch B 25/50/100 μM, co-treatment with APAP for 48 h	PPP: G6PD↑, PAK4↑, PLK1↑ (NADPH antioxidant capacity↑); MAPK: p-P38↓/p-JNK↓/p-ERK↓; apoptosis proteins: Bcl-2↑, Bax↓, cleaved caspase-3↓, PARP cleavage↓; ROS and inflammatory factors↓	Significantly improved cell viability under APAP injury and suppressed apoptosis, oxidative stress, and inflammation
Zhang et al. (2019)	*In vivo*: male Wistar rats, CCl_4_-induced liver fibrosis	Sch B 25 or 50 mg/kg/day, gavage, co-treatment once daily for 5–8 weeks during CCl_4_ exposure	Oxidative stress: MDA↓, SOD↑, GSH↑, GSH-Px↑; ER stress: ATF4/ATF6/CHOP↓; apoptosis: TUNEL↓, Tp53↓; CYP2E1/CYP1A2 modulation; PPARα/β/γ restored	Significantly improved liver function and attenuated fibrosis and collagen deposition
Zhang et al. (2015)	*In vitro*: AML-12 hepatocytes; RAW264.7 macrophages	Sch B 0.1–25 μM, treatment for 24 h (viability testing up to 200 μM)	Cell cycle: CDK2↓, Cyclin D1↓; p27 Kip1↑, Chk1↑; mitochondrial apoptosis: Bcl-2/Bcl-xl↓, Bax↑, Cyt c release↑, caspase-3/PARP cleavage↑; autophagy: PI3K/Akt/mTOR phosphorylation↓	Inhibited cell proliferation and induced apoptosis and autophagy-related responses
Xu et al. (2024)	*In vivo*: BALB/c mouse renal I/R (45min +24 h) *In vitro*: HK-2 H/R	*In vivo*: Sch B 20 or 40 mg/kg/day, gavage, pretreatment for 7 days *In vitro*: Sch B 20 μM, pretreatment for 12 h	PI3K/Akt: p-AKT restored; mitochondrial dysfunction attenuated: mitochondrial superoxide↓, ΔΨm restored; oxidative stress and apoptosis-related indices decreased	Significantly alleviated renal I/R injury and improved renal function and cell survival
Liu et al. (2011)	*In vivo*: Wistar rats (both sexes, equal numbers), HgCl_2_-induced nephrotoxicity	Sch B 16 mg/kg/day, gavage, pretreatment for 2 days before HgCl_2_	Oxidative stress: ROS↓, MDA↓; antioxidant defense: GSH↑, SOD↑, GSH-Px↑; improved mitochondrial ultrastructural damage (TEM); apoptosis reduced	Attenuated HgCl_2_-induced nephrotoxicity and preserved tubular structural integrity
Liu et al. (2023)	*In vivo*: db/db mouse DKD *In vitro*: HK-2 high glucose (30 mM)	*In vivo*: Sch B 50 mg/kg/day, gavage, treatment once daily for 6 weeks *In vitro*: Sch B 10–40 μM for 48 h	Mitochondrial function: ΔΨm↑, ROS↓, ATP↑; biogenesis/fusion: PGC-1α↑, TFAM↑, MFN1/2↑; key mediator: KCP↑; pathways: PI3K/Akt↓, AMPK↑	Improved DKD progression and suppressed tubular EMT and renal interstitial fibrosis
Zhao et al. (2018)	*In vivo*: male SD rats, myocardial I/R (45min +24 h)	Sch B 60 mg/kg/day, gavage, pretreatment once daily for 15 days before I/R LY294002 0.3 mg/kg i.p. for validation	PI3K/Akt: p-Akt↑; mitochondrial apoptosis: Bax/Bcl-2 ratio↓, cleaved caspase-3↓; LY294002 abolished protection	Significantly reduced infarct size and decreased cardiomyocyte death
Chen et al. (2013)	*In vivo*: C57BL/6J mouse MI (permanent LAD ligation) *In vitro*: H9c2 hypoxia model	*In vivo*: Sch B 80 mg/kg/day, gavage, treatment once daily for 3 weeks *In vitro*: Sch B 5, 10, and 20 μM, pretreatment for 16 h	Anti-inflammatory: NF-κB↓, TGF-β1↓, TNF-α↓; anti-apoptotic: Bcl-2↑, Bax↓, ASK1↓; eNOS: p-eNOS↑; repair factors: GATA4↑	Improved post-MI survival and cardiac function and reduced inflammation and fibrosis
Ai et al. (2019)	*In vivo*: C57BL/6 mice, TAC (4 weeks) *In vitro*: H9c2 + Ang II (1 μM)	*In vivo*: Sch B 80 mg/kg/day, gavage, treatment once daily for 4 weeks *In vitro*: Sch B 5, 10, and 20 μM, pretreatment for 16 h	MAPK: p-ERK1/2↓, p-JNK1/2↓, p-P38↓; hypertrophy/fibrosis markers (ANP/BNP/β-MHC/CTGF/Collagen)↓	Attenuated pressure overload–induced hypertrophy and fibrosis and improved cardiac function
Misgeld and Schwarz (2017)	*In vivo*: SD rats, THP cardiotoxicity (8 weeks) *In vitro*: H9c2 THP injury	*In vivo*: Sch B 50 mg/kg/day, dietary administration (500 mg/kg chow, 0.5‰), co-treatment for 8 weeks *In vitro*: Sch B 50 μM, pretreatment for 2 h followed by co-treatment with THP	mPTP opening↓ (mitochondrial swelling/Calcein-AM); ROS↓; mitochondrial apoptosis: mitochondrial Bax↓, mitochondrial Cyt c retention↑, cytosolic Cyt c↓, cleaved caspase-9/3↓; similar to CsA	Markedly alleviated anthracycline cardiotoxicity and improved cardiac function and myocardial structure
Tang et al. (2022)	*In vivo*: SD rats, THP cardiotoxicity (7 weeks) *In vitro*: neonatal rat primary cardiomyocytes	*In vivo*: Sch B 50 mg/kg/day, dietary administration (500 mg/kg chow, 0.5‰), co-treatment for 7 weeks *In vitro*: Sch B 50 μM, pretreatment for 2 h followed by co-treatment with THP for 12 h	Antioxidant: ROS↓, MDA↓; SOD/CAT/T-AOC↑; NOX2↓; apoptosis proteins: Bcl-2/Bax ratio↑, cleaved caspase-3↓	Improved anthracycline cardiotoxicity-induced cardiac dysfunction and reduced cardiomyocyte death
Lee et al. (2012)	*In vivo*: male SD rats, MCAo cerebral I/R (120min +24 h)	Sch B 10 or 30 mg/kg, intraperitoneal injection, administered 30 min before ischemia and 2 h after reperfusion	Inflammation: TNF-α↓, IL-1β↓; microglial activation↓ (OX-42/CD11b); matrix degradation: MMP-9 activity/expression↓	Significantly reduced infarct volume and improved neurological scores
Ba et al. (2015)	*In vivo*: C57BL/6 mice, 6-OHDA PD model *In vitro*: SH-SY5Y 6-OHDA injury	*In vivo*: Sch B 80 mg/kg/day, gavage, pretreatment once daily for 7 days *In vitro*: Sch B 100 μM, pretreatment for 2 h	miR-34a↓; Nrf2↑ (released from miR-34a suppression); HO-1↑, NQO1↑; miR-34a mimic reversed the effects	Improved dopaminergic neuron survival and alleviated Parkinsonian motor deficits
Giridharan et al. (2015)	*In vivo*: male SD rats, ICV infusion of Aβ_1_–_40_ (300 pmol/day ×14 days)	Sch B 25 or 50 mg/kg/day, gavage, treatment once daily from day 3 to day 28	RAGE/NF-κB/p38-ERK↓; oxidative/nitrosative stress reduced; HSP70↑; Beclin-1 moderately↑; neuronal apoptosis-related indices decreased	Reduced cerebral Aβ burden and improved learning and memory
Jiang et al. (2015)	*In vitro*: PC12 cells, H_2_O_2_ (200 μM, 24 h)-induced injury	Sch B 2.5, 5, 10 μM, pretreatment for 2 h	PI3K/Akt: p-Akt↑; antioxidant: ROS↓, MDA↓, SOD↑; mitochondrial apoptosis: Bcl-2/Bax ratio↑; LY294002 blocked anti-apoptotic effects	Markedly improved cell survival and reduced cell death under oxidative injury
Yang et al. (2024a)	*In vitro*: BV2 microglia, LPS (1 μg/mL)-induced inflammation/M1 polarization	Sch B 12.5, 25, 50 μM, pretreatment 3 h and co-treatment with LPS for 21 h	miR-124↑; TLR4/MyD88/NF-κB↓ (IKK-α↓, p65↓, IκB-α↑); MAPK: p-p38↓/p-ERK↓/p-JNK↓; miR-124 inhibition reversed effects	Suppressed inflammation and promoted microglial shift toward an anti-inflammatory phenotype
Lam and Ko (2012)	In vitro: differentiated rat PC12 neuronal cells; 3-NP-induced HD-like cytotoxicity model	(−)Sch B 5 or 15 μM, pretreatment for 6 h followed by post-treatment for 16 h	GSH depletion↓; JNK phosphorylation↓ → PDH inhibition↓ (PDH activity↑)	Cell necrosis and mitochondria-dependent apoptosis were reduced
Leong et al. (2014)	*In vitro*: SH-SY5Y cells, Aβ_25_–_35_ (40 μM, 48 h)-induced apoptosis	(−)Sch B 5 or 15 μM, pretreatment for 6 h, washout 16 h, then Aβ exposure	Glutathione cycling: GSH↑, reduced Aβ-induced GSH depletion; G6PDH↑, GR↑; Nrf2 activation↑; tau hyperphosphorylation↓	Reduced Aβ-induced neuronal apoptosis and preserved antioxidant capacity
Cai et al. (2016)	*In vivo*: male BALB/c mice, LPS-induced ALI (intratracheal LPS 1 mg/kg)	Sch B 25, 50, and 75 mg/kg, gavage, single pretreatment 1 h before LPS administration	P2X7↓; MyD88↓; IκBα/p-p65 phosphorylation↓; TNF-α/IL-1β/IL-6↓	Attenuated ALI and improved pulmonary inflammation and edema
Chen et al. (2021a)	*In vivo*: male BALB/c mice, OVA-induced allergic asthma (chronic sensitization + challenge)	Sch B 15, 30, and 60 mg/kg, gavage, pretreatment 1 h before each allergen challenge	NF-κB: p-IKKα↓, p-p65↓, p-IκBα↓; Nrf2/HO-1↑; oxidative stress: ROS/NO/MDA↓, SOD/CAT/GSH-Px↑	Significantly alleviated asthma phenotypes and reduced airway inflammation and hyperresponsiveness
Chen et al. (2021b)	*In vivo*: female SD rats, OVA asthma (8 weeks); *In vitro*: primary alveolar macrophages, LPS inflammation	*In vivo*: Sch B 80 mg/kg/day, gavage, treatment once daily *In vitro*: Sch B 100 μM, co-treatment with LPS	Inflammasome: NLRP3↓, caspase-1↓, GSDMD-N↓; pyroptosis: LDH release↓; miR-135a-5p↑ → TRPC1↓; STAT3/NF-κB phosphorylation↓	Reduced airway inflammation and remodeling and suppressed inflammasome-related inflammation
Jia et al. (2017)	*In vivo*: male C57BL/6 mice, cigarette-smoke–induced acute airway inflammation (5 days)	Sch B 20, 40, and 80 mg/kg, intraperitoneal injection, pretreatment 2 h before each cigarette smoke exposure	Nrf2/HO-1↑; NF-κB activation↓ (p-p65↓, p-IκBα↓); MPO/MDA↓; SOD/GSH↑; TNF-α/IL-1β/IL-6↓; iNOS/COX-2↓	Attenuated smoke-induced airway inflammation and improved lung injury
Wang et al. (2020)	*In vivo*: male ICR mice, BLM-induced pulmonary fibrosis (intratracheal 5 mg/kg; 28 days)	Sch B 5, 10, and 20 mg/kg/day, gavage, treatment once daily for 28 days after BLM	Wnt/β-catenin↓; MMP7↓; antioxidant capacity↑ (SOD↑, T-AOC↑); TGF-β1↓	Markedly reduced pulmonary fibrosis and collagen deposition and improved lung structural damage
Yang et al. (2024b)	*In vivo*: male C57BL/6 mice, BLM-induced pulmonary fibrosis (intratracheal 2 mg/kg)	Sch B 3 mg/kg/day, gavage, treatment once daily for 7 days after BLM	Autophagy: Beclin-1↑, LC3-II↑ (blocked by CQ); AKT–mTOR phosphorylation↓ (reversed by 3-MA); myofibroblast activation: α-SMA↓, Collagen I/III↓, CTGF↓, FN↓	Inhibited fibrosis progression and improved lung function; effects depended on enhanced autophagy
Wu et al. (2017b)	*In vitro*: HPASMCs, hypoxia (1% O_2_, 24 h)-induced PAH phenotype	Sch B 50 μM, treatment for 24 h (under hypoxia)	TGF-β1↓; NOX4↓, ROS↓, MMP-2↓; MAPK: p-ERK1/2↓, p-p38↓; α-SMA↓; mitochondria-related apoptosis: Bcl-2↓, Bax↑, caspase-3↑	Suppressed hypoxia-induced abnormal proliferation and migration and promoted apoptosis
Co et al. (2024a)	*In vivo*: HCT116 xenografts in nude mice *In vitro*: human colorectal cancer cell lines (e.g., HCT116)	*In vivo*: Sch B 50 mg/kg, gavage, administered every other day for 14 days *In vitro*: Sch B 25–100 μM, treatment for 48 h	Cell cycle: G0/G1 arrest↑; apoptosis: BAX↑, cleaved caspase-3↑, BAX/BCL-2 ratio↑; ER stress-UPR: CHOP (DDIT3)↑; CHOP siRNA partially reversed effects	Inhibited colorectal cancer growth and induced cell death; significantly suppressed tumor growth *in vivo*
Fang et al. (2025)	*In vitro*: nasopharyngeal carcinoma cells and 3D organoids, radiotherapy model	Sch B 10–40 μM, pretreatment for 4–6 h before irradiation	Direct targeting of CDK4/6 (CETSA: increased thermal stability); CDK4/6 protein↓; G1 arrest↑; delayed DNA damage repair (γ-H2AX foci↑)	Enhanced radiosensitivity and increased radiotherapy-induced cytotoxicity
Yang et al. (2016)	*In vivo*: HCCC-9810 xenografts in nude mice *In vitro*: cholangiocarcinoma cells (HCCC-9810, RBE)	*In vivo*: Sch B 20 or 80 mg/kg, intraperitoneal injection, administered every 2 days for 30 days *In vitro*: Sch B 20, 40, and 80 μM, treatment for 48 h	Cell cycle: Cyclin D1↓, CDK4↓; mitochondrial apoptosis: ΔΨm↓, Bax↑, Bcl-2↓, cleaved caspase-9/3↑, PARP cleavage↑	Inhibited cholangiocarcinoma cell proliferation and induced apoptosis; significantly suppressed tumor growth *in vivo*
Wang et al. (2017)	*In vitro*: DOX-resistant breast/ovarian cancer cells	Sch B 5–20 μM, pretreatment for 12 h followed by co-treatment with DOX for 48 h	P-gp expression/activity↓ → intracellular DOX accumulation↑; survivin↓ (proteasomal degradation↑); 26S proteasome activity↑; MG-132 blocked survivin downregulation	Restored DOX sensitivity and enhanced chemotherapy-induced cell death in resistant cells
Nasser et al. (2019)	*In vitro*: prostate cancer cells (LNCaP and DU145)	Sch B 12.5, 25, and 50 μM, treatment for 48 h	ROS↑ (blocked by N-acetyl-L-cysteine (NAC)); mitochondrial apoptosis: ΔΨm↓, Cyt c release↑, Bax↑, Bcl-2↓, cleaved caspase-3↑, PARP cleavage↑; PI3K/Akt↓; JAK2/STAT3↓; androgen receptor (AR) transcriptional activity↓; S-phase arrest↑	Inhibited prostate cancer cell growth and induced pronounced apoptosis, while suppressing IL-6–driven proliferative signaling
Lv et al. (2015)	*In vitro*: A549 lung adenocarcinoma cells	Sch B 12.5, 25, and 50 μM, treatment for 72 h	Cell cycle: Cyclin D1/CDK4/CDK6↓, p53↑, p21↑; mitochondrial apoptosis: Bax↑, Cyt c↑, cleaved caspase-9/3↑, Bcl-2↓; migration/invasion: HIF-1α↓, VEGF↓, MMP-2/9↓	Inhibited proliferation and induced apoptosis, while suppressing migration and invasion
Hu et al. (2025)	*In vitro*: HT22 hippocampal neuronal cells, H_2_O_2_ (100 μM, 24 h)-induced injury	Sch B 1.25 μM, pretreatment for 24 h before oxidative stress	Sirt3↑; mitochondrial dynamics: MFN1/MFN2↑, DRP1/FIS1↓; mitochondrial function: ΔΨm↑, ATP↑, ROS↓; mitochondrial structural integrity preserved (TEM)	Improved neuronal viability under oxidative injury and reduced cell death
Hu et al. (2024)	*In vitro*: C28I2 chondrocytes, LPS (10 μg/mL, 24 h)-induced mitochondrial damage	Sch B 0.05 μM, co-treatment for 24 h	Mitochondrial dynamics: MFN1/MFN2/OPA1↑, DRP1↓; mitochondrial function: ΔΨm↑ (JC-1), mitochondrial activity↑; apoptosis: caspase-3↓	Attenuated inflammation-induced mitochondrial damage and improved chondrocyte viability and migration
Xu et al. (2025)	*In vivo*: C57BL/6 mouse renal I/R (30min +24 h); *In vitro*: HK-2 H/R	*In vivo*: Sch B 20 mg/kg, intraperitoneal injection, single pretreatment 1 h before I/R *In vitro*: Sch B 10 μM, pretreatment for 12 h	AKT1: p-AKT1↑; mitochondrial enrichment↑; dynamics: MFN1/MFN2↑, DRP1↓; function: ΔΨm↑, ATP↑, ROS↓; AKT1 inhibition weakened effects	Significantly alleviated renal I/R injury and reduced tubular apoptosis
Y et al. (2022)	*In vivo*: C57BL/6J mice, HFD-NAFLD; *In vitro*: HepG2/primary hepatocytes, FFA-induced steatosis	*In vivo*: Sch B 50 mg/kg/day, gavage, treatment once daily for 5 weeks *In vitro*: Sch B 12.5–50 μM, treatment for 24 h	AMPK↑/mTOR↓; autophagic flux↑ (LC3B-II↑, p62↓, LAMP1↑); TFEB nuclear translocation↑; lipophagy↑; FAO/ketogenesis enzymes (CPT1A/ACOX1/ACADL/ACADM/HMGCS2)↑	Reduced hepatic lipid droplet accumulation and improved lipid metabolic dysregulation; decreased steatosis *in vivo*
Tao et al. (2021)	*In vivo*: C57BL/6 mice, acute ethanol-induced myocardial injury; *In vitro*: H9c2/primary cardiomyocytes, ethanol injury	*In vivo*: Sch B 800 mg/kg/day, gavage, co-treatment once daily for 3 consecutive days *In vitro*: Sch B 100–1,000 μM, pretreatment before ethanol exposure	NOX4↓ → ROS↓; autophagy: LC3-II↓, Beclin-1↓, p62↓; apoptosis: cleaved caspase-3↓; NOX4 siRNA blocked ROS/autophagy/apoptosis changes	Improved ethanol-induced myocardial injury and maintained cardiac functional stability
Li et al. (2023a)	*In vivo*: ICR mice, APAP-induced acute liver injury; *In vitro*: HepG2, APAP injury	*In vivo*:Sch B 50 mg/kg/day, gavage, pretreatment once daily for 7 days before APAP *In vitro*: Sch B 20 μM, pretreatment for 12 h	EGFR/p-EGFR↓; TFEB↑; autophagy: LC3↑, Beclin-1↑; inflammatory factors (TNF-α/IL-1β)↓; EGFR–TFEB–autophagy axis mediated protection	Attenuated APAP-induced hepatocyte injury and reduced hepatic inflammation
Lai et al. (2017)	*In vitro*: HK-2 cells, CsA (10 μM, 24 h)-induced nephrotoxicity	Sch B 2.5, 5, and 10 μM, pretreatment for 12 h	ROS↓, GSH↑, ΔΨm↑; Nrf2 nuclear translocation↑ → HO-1/NQO1/GCLM↑; mitochondrial apoptosis: Bax↓, Bcl-2↑; restored impaired autophagic flux (adjusted LC3-II/Beclin-1/p62)	Attenuated CsA-induced tubular injury and improved cell survival
Niu et al. (2025)	*In vivo*: C57BL/6J mice, DCM (HFD + STZ) *In vitro*: H9c2 high glucose + palmitate injury	*In vivo*: Sch B 12.5 or 25 mg/kg/day, gavage, treatment once daily for 10 weeks *In vitro*: Sch B 12.5–50 μM, treatment for 72 h	Ferroptosis: Fe^2+^↓, p53↓, SLC7A11↑, GPX4↑; mitochondrial function: ΔΨm↑, ATP↑, respiration↑, ROS↓; fatty acid β-oxidation↑, TCA intermediates↑; erastin reversed effects; no additive effect with ferrostatin-1	Improved cardiac function in DCM and alleviated myocardial injury and metabolic dysregulation
He et al. (2024)	*In vivo*: male SD rats, BCAO vascular dementia model	Sch B 10, 20, and 40 mg/kg/day, gavage, treatment once daily for 14 days	Hypoxia-related: HIF-1α↓; mitochondrial transport-related: TRAK2↓, HUMMR↓	Improved learning and memory and reduced hippocampal neuronal injury
Chiu et al. (2008)	*In vivo*: female SD rats, gentamicin-induced nephrotoxicity	Sch B 1 or 10 mg/kg/day, gavage, pretreatment once daily for 15 days before gentamicin administration	Renal mitochondrial antioxidant defense: mtGSH↑, α-tocopherol↑, Mn-SOD↑; mitochondrial function: ATP-generating capacity↑; mitochondrial homeostasis: Ca^2+^ burden↓, mPT sensitivity↓, Cyt c release↓	Significantly attenuated gentamicin nephrotoxicity and preserved mitochondrial bioenergetic stability
Chen et al. (2008)	*In vivo*: female SD rats, cerebral I/R (bilateral common carotid artery occlusion 120 min + reperfusion 60 min)	Sch B 1, 10, or 30 mg/kg/day, gavage, pretreatment once daily for 15 days before ischemia induction	Brain mitochondrial antioxidant defense: mtGSH↑, α-tocopherol↑, Mn-SOD↑; mitochondrial homeostasis: lipid peroxidation↓, Ca^2+^ burden↓, mPT sensitivity↓, Cyt c release↓	Significantly attenuated cerebral I/R injury and improved survival of ischemic tissue
Wu et al. (2019)	*In vivo*: male C57BL/6 mice, forced-swim–induced acute stress/anxiety-like behavior	Sch B 30 or 60 mg/kg, gavage, pretreatment twice daily for 3 consecutive days before I/R	Nrf2↑, Keap1↓; antioxidant: SOD↑, GSH↑; oxidative stress: ROS↓, MDA↓	Alleviated stress-induced anxiety-like behaviors and protected amygdala neurons
Piao et al. (2021)	*In vitro*: primary hippocampal neurons from neonatal SD rats, Aβ_1_–_42_ (1 μM, 48 h)-induced mitochondrial damage	Sch B 2 μg/mL, pretreatment for 1 h followed by co-treatment with Aβ	mPTP opening↓; ΔΨm↑; Cyt c release↓; energy metabolism: ATP↑, CS activity↑, glycolytic enzymes↑; biogenesis: PGC-1α↑, mitochondrial mass↑; dynamics: Mfn1/2↑, Opa1↑, Drp1↓	Reversed Aβ-induced mitochondrial dysfunction and improved neuronal survival
Ko et al. (2008)	*In vivo*: C57BL/6J mice, natural aging model (multi-tissue)	Sch B 120 mg/kg chow (0.012% w/w), dietary administration, continuous treatment from 36 to 120 weeks of age	Antioxidant: mtGSH↑, α-tocopherol↑, Mn-SOD↑; mitochondrial ROS production↓; ATP-generating capacity↑	Delayed multi-tissue age-related mitochondrial decline and improved overall survival outcomes
Chiu et al. (2011)	*In vivo*: perfused heart I/R (tissue collection) *In vitro*: H9c2 H/R	*In vivo*: Sch B 481 mg/kg, gavage, single pretreatment 48 h before heart isolation for I/R *In vitro*: (−)Sch B 15 μM, pretreatment for 6 h	CYP-mediated ROS → ERK↑ → Nrf2↑; GCLm↑, GR↑, G6PDH↑, Trx-1↑, GSH↑; ERK/Nrf2 inhibition abolished protection	Reduced H/R-induced cardiomyocyte injury and improved cardiac I/R outcomes
Shi et al. (2021)	*In vivo*: SD rats, THP cardiotoxicity (8 weeks) *In vitro*: H9c2 THP injury	*In vivo*: Sch B 50 mg/kg/day, dietary administration (500 mg/kg chow, 0.5‰), co-treatment once daily for 8 weeks *In vitro*: Sch B 50 μM, pretreatment for 2 h followed by co-treatment with THP for 12 h	mPTP opening↓; mitochondrial Ca^2+^ overload↓; ROS↓; Cyt c release↓ → caspase-9/3↓; mechanism associated with mtGSH improvement	Markedly attenuated anthracycline-induced cardiotoxicity and improved cardiac function
Chiu et al. (2007)	*Ex vivo*: perfused rat hearts I/R (ischemia 40 min + reperfusion 20 min; hearts collected after *in vivo* dosing)	Sch B 400–801 mg/kg, gavage, single pretreatment 48 h before heart isolation for I/R	Reduced Ca^2+^-triggered mPTP sensitivity; decreased mitochondrial Ca^2+^ overload; ROS production↓; Cyt c release↓ (pattern similar to CsA)	Significantly reduced I/R-induced myocardial injury and improved cardiomyocyte survival

Mechanistic arrows (↑/↓) indicate up- or downregulation relative to the injury/control condition in each study. Pretreatment refers to Sch B exposure prior to injury induction; co-treatment indicates concurrent exposure with the insult; treatment indicates administration after injury induction. Although Table 1 provides representative evidence linking Schisandrin B to mitochondria-associated outcomes across organ injury models, important limitations should be acknowledged. First, many studies rely on acute experimental systems or simplified *in vitro* models, which are valuable for mechanistic plausibility but cannot on their own substantiate organ-level pharmacological claims. Second, dosing regimens and concentration ranges are heterogeneous, and minimal effective concentrations or dose–response relationships are not consistently established, limiting pharmacological extrapolation. Third, the methodological quality of the literature varies, with inconsistent use of positive controls, limited validation of causal mitochondrial endpoints, and frequent reliance on indirect oxidative stress or apoptosis markers. Finally, reporting of material definition (e.g., purity, source, and batch characterization) remains incomplete in some studies, emphasizing the need for improved standardization. Future research priorities should therefore include more rigorous pharmacological design with appropriate controls, clinically relevant exposure ranges, standardized mitochondrial functional assays, and quantitative assessment of intramitochondrial exposure to strengthen translational interpretation.

Note:
*In vivo* doses are uniformly expressed in mg/kg.
*In vitro* concentrations are uniformly expressed in μM.
Values originally reported in mmol/kg or g/kg were converted based on the molecular weight of Sch B (400.46 g/mol).

## 5 A “unified mechanistic model” across multiple organs

Integrating the evidence summarized in Section 4 suggests that the mitochondria-related effects of Sch B across multiple organ systems share certain biological commonalities, rather than being entirely isolated observations. Although the liver, kidney, heart, lung, nervous system, and tumor system differ significantly in structural and functional features, substrate utilization patterns, and dominant pathological drivers, these organs often exhibit similar types of mitochondrial abnormalities during early injury stages or disease progression across diverse models. Such alterations include reduced OXPHOS efficiency, limited ATP generation, accumulation of mtROS, impaired ΔΨm and cristae structure, disrupted dynamic balance, and disturbed mitochondrial quality control. Available evidence indicates that these changes often occur at relatively upstream positions within pathological cascades and may contribute to determining whether organ injury progresses toward irreversible stages. In this context, Sch B appears to exert its multi-organ actions not through highly specific regulation of a single signaling pathway, but rather through the preservation of overall mitochondrial functional stability. Specifically, its effects under different pathological pressures often manifest as delaying the onset or progression of mitochondrial imbalance, enabling cells to maintain a relatively stable metabolic state during acute insults or sustained stress. Given the reproducibility of these phenomena across multiple organs and experimental models, it can be inferred that the multi-organ effects of Sch B are primarily based on integrated regulation at the level of mitochondrial homeostasis, rather than a simple superposition of multiple independent mechanisms.

### 5.1 Key shared targets across multiple organs

From a cross-organ perspective, current studies suggest that the repeatedly affected targets of Sch B across different systems are not single molecular nodes, but rather several functional layers involved in maintaining mitochondrial homeostasis. Under acute injury or abrupt metabolic stress, highly mitochondria-dependent cell types typically experience rapid ATP decline and ΔΨm instability, which are closely linked to necrosis or mitochondria-dependent apoptosis. Acute liver injury, renal I/R, myocardial I/R, and cerebral I/R models consistently indicate that Sch B treatment slows early progression of energy metabolic disturbance and partially preserves OXPHOS function, correlating with improved cellular injury outcomes (Cheng et al., 2022; Xu et al., 2024; Chiu et al., 2007; Chen et al., 2008). Notably, this effect can be more accurately described as delaying or buffering the process of energy imbalance, rather than directly blocking the injurious factor itself.

In models dominated by oxidative stress and free radical-mediated injury, the mitochondrial membrane system and cristae structure are often affected early on. Persistent lipid peroxidation disrupts the membrane microenvironment required for respiratory chain proteins and is accompanied by ΔΨm decline and reduced respiratory efficiency. Studies in hepatotoxicity, drug-induced nephrotoxicity, drug-induced cardiotoxicity, and infection-related ALI consistently show that Sch B reduces lipid peroxidation-related indicators and delays the extent of mitochondrial membrane structural damage, thereby preserving mitochondrial functional output under high oxidative pressure (Zhang et al., 2019; Liu et al., 2011; Shi et al., 2021; Cai et al., 2016). However, while these findings suggest mitochondrial protection, the full extent of mitochondrial dysfunction and the direct role of Sch B in mitigating these changes requires further mechanistic clarity.

Under chronic metabolic load or long-term inflammatory contexts, mitochondrial injury often manifests not as acute functional collapse but as progressive accumulation of functionally impaired mitochondria, increased network fragmentation, and insufficient renewal. Models of NAFLD/NASH, ALD, DKD, HF, neurodegenerative diseases, and chronic lung injury consistently reflect this trajectory (Leong and Ko, 2016; Liu et al., 2023; Ai et al., 2019; Monzio Compagnoni et al., 2020). In these models, the effects of Sch B are more closely associated with the preservation of mitochondrial quality control continuity, including slowing the decline in the proportion of functional mitochondria and modulating dynamic balance, which may contribute to delaying organ functional deterioration.

In addition, the interaction between mitochondrial dysfunction and inflammatory responses is a recurring phenomenon in multi-organ injury. mtROS generation and mtDNA release can activate inflammation-related signaling pathways, while inflammation can exacerbate mitochondrial damage. This process has been observed in hepatic inflammation and fibrosis, neuroinflammation, and ALI models (Wu X. et al., 2017; Willemen et al., 2023; Zhan and Shen, 2022). Observations across multiple models showing associations between Sch B treatment and reduced inflammatory cytokine levels suggest that Sch B may indirectly influence tissue injury severity by attenuating mitochondria-associated inflammatory drivers (Lee et al., 2012; Yang Y. et al., 2024; Cai et al., 2016; Zhu et al., 2023). However, it is important to note that inflammation is multifaceted, and the role of Sch B in modulating both mitochondrial function and inflammatory cascades needs further mechanistic elucidation.

It should be emphasized that the above effects show clear dependence on dose, time window, and cellular context. Existing studies indicate that under specific experimental conditions, Sch B may also induce cell cycle arrest, mitochondria-dependent apoptosis, or autophagy-related alterations, suggesting that its mitochondrial regulatory actions do not invariably lead to protective outcomes. Therefore, within a unified mechanistic framework, it is necessary to incorporate its potential biological boundaries and safety considerations.

### 5.2 Differential regulation across organs

Although Sch B involves a relatively conserved set of mitochondrial homeostasis processes across multiple organ systems, evidence suggests that its functional emphasis in different organs is more closely related to the mitochondrial functional layer most disrupted during organ-specific pathological progression, rather than reflecting fundamental differences in the mechanism of action itself. In other words, the regulatory effects of Sch B likely target the mitochondrial functional layer that constitutes the limiting factor in a given pathological context.

Under different pathological conditions, mitochondrial imbalance does not occur simultaneously across all dimensions such as energy production, structural integrity, and quality control, but often exhibits certain temporal ordering or relative dominance. For example, under high-intensity stress such as acute ischemia–reperfusion or toxin exposure, injury progression in some organs is largely associated with rapid interruption of energy supply, making ATP decline a key limiting determinant of cell fate. In contexts dominated by oxidative stress or hyperoxia-associated inflammation, mitochondrial membrane system and cristae structural injury may engage earlier in the imbalance process. Under chronic metabolic load or long-term inflammation, mitochondrial dysfunction more frequently manifests as insufficient renewal and gradual loss of functional mitochondria rather than immediate functional loss (Jaeschke et al., 2012; Zhang et al., 2019; Tao et al., 2021; Ai et al., 2019). Within this framework, the differential effects of Sch B can be interpreted as follows: Sch B does not equally strengthen all components of mitochondrial homeostasis under all conditions, but instead influences the functional layer disrupted earliest or most prominently in a given pathological context. Accordingly, in models driven primarily by acute energy imbalance, its effects are more related to preservation of OXPHOS and stabilization of ΔΨm; in models driven by oxidative injury and membrane disruption, its effects are reflected by reduced lipid peroxidation and alleviated structural damage; and in chronic metabolic diseases, its actions are mainly associated with continuity of mitochondrial renewal and metabolic coordination (Xu et al., 2024; Zhang et al., 2019; Liu et al., 2023; Monzio Compagnoni et al., 2020). These shifts in functional emphasis are likely reflective of differential manifestations of the same regulatory capacity under distinct imbalance backgrounds.

Moreover, intrinsic metabolic architecture and energy supply modes of different organs determine the degree of dependence on specific mitochondrial functional dimensions, shaping how Sch B effects are expressed. High-energy flux organs are more sensitive to continuous mitochondrial energy supply; organs with pronounced oxygen gradients or perfusion dependence are more vulnerable to mitochondrial disruption under oxygen fluctuations; organs chronically exposed to high oxygen are more prone to accumulating oxidative pressure under inflammation; and in tumor systems, mitochondrial homeostasis itself directly participates in stress adaptation and therapeutic tolerance (Zhang et al., 2023; Zhang et al., 2021; Che et al., 2014; Bisaccia et al., 2021; Schumacker et al., 2014; Liu and Shi, 2020; Chen et al., 2024). The distinct effects of Sch B across these systems may therefore represent concrete manifestations of its mitochondrial regulation under different metabolic constraint conditions.

In addition, organ differences are also related to whether mitochondrial homeostasis disruption leads to structural remodeling. In some organs, mitochondrial dysfunction is frequently accompanied by fibrosis, vascular remodeling, or tissue stiffening; in the nervous system, it manifests as axonal degeneration and synaptic functional loss; and in tumor systems, mitochondrial homeostasis directly regulates cell survival thresholds and treatment responses (Li et al., 2021; Wang et al., 2020; Wu J. et al., 2017; Yang et al., 2016). Accordingly, differences in the protective, delaying, or sensitizing effects of Sch B across organs are likely related to the distinct biological functions that mitochondrial homeostasis serves in different tissues.

In summary, available evidence supports the interpretation that differential effects of Sch B across organs do not reflect fundamental mechanistic divergence, but rather are shaped by organ-specific metabolic architecture, dominant pathological pressures, and the key limiting layers of mitochondrial dysfunction under those contexts. Within a unified mitochondrial homeostasis regulation framework, these differences are primarily manifested as shifts in regulatory emphasis, providing a more cautious theoretical basis for evaluating its potential applications from the perspective of pathological type rather than organ classification alone.

## 6 Drug development prospects and challenges of Sch B

As a lignan metabolite with a clearly defined botanical origin and well-characterized chemical structure, Sch B has been reported to exert mitochondria-associated protective phenotypes in multiple organ injury and disease models, encompassing the liver, kidney, heart, lung, nervous, and immune systems—tissues that are highly dependent on mitochondrial homeostasis. This cross-organ and cross-model profile provides a conceptual basis for considering Sch B as a candidate compound for further exploration within the framework of “mitochondrial homeostasis regulation”. However, it should be emphasized that translation from mechanistic pharmacology into genuine drug development remains highly challenging and requires systematic resolution of several key issues that are still insufficiently validated, including adequate *in vivo* exposure, delivery and targeting efficiency, safety boundaries, and precise identification of clinical indications (Murphy and Hartley, 2018).

From a pharmacokinetic perspective, the strong lipophilicity of Sch B is a physicochemical property that may facilitate membrane permeation, yet also introduces typical limitations such as low aqueous solubility, pronounced first-pass metabolism, and high plasma protein binding. Available studies indicate that Sch B can be absorbed after oral administration and exhibits a relatively large apparent volume of distribution, with detectable tissue accumulation particularly in metabolically active organs such as the liver (Liang et al., 2021). Nevertheless, most PK studies remain limited to plasma or total tissue concentration measurements, and direct quantitative evidence is lacking for its subcellular exposure—especially its actual intramitochondrial exposure profile (e.g., free intramitochondrial concentration, retention time, and dynamic distribution) (Ehambarampillai and Wan, 2025). This implies that, although many animal studies have observed mitochondria-related pharmacodynamic phenotypes of Sch B, its “mitochondrial pharmacokinetic properties” remain largely inferred indirectly. For candidate compounds in which mitochondria are proposed as a key functional site, this information gap becomes particularly critical during drug development. Future work should integrate mitochondrial isolation techniques, subcellular quantitative analyses, and tracer-labeling approaches to establish more rigorous PK/PD linkage models for Sch B, thereby defining its effective exposure range, dose–response relationship, and potential therapeutic window.

To address the bottleneck of limited solubility and suboptimal delivery efficiency, recent studies have attempted to improve the *in vivo* behavioral profile of Sch B using pharmaceutic approaches. Encapsulation of Sch B into liposomes, solid lipid nanoparticles, or polymeric nanocarriers has been reported to enhance its aqueous dispersibility, *in vivo* stability, and pharmacodynamic performance in certain experimental disease models (M et al., 2025). On this basis, incorporation of mitochondrial-targeted delivery strategies is sometimes proposed as potentially advantageous. Lipophilic cations represented by triphenylphosphonium (TPP^+^) can accumulate in the inner mitochondrial membrane in a ΔΨm-dependent manner and have been widely used to construct mitochondria-directed molecules and nanomedicines, such as MitoQ and MitoTEMPO. In theory, combining Sch B with such mitochondrial-targeting delivery systems—for example, constructing TPP^+^-modified Sch B nanocarriers—may increase local mitochondrial exposure, thereby potentially enhancing efficacy while reducing systemic dosing requirements (Han et al., 2019; Rondeau et al., 2024; Gruber et al., 2013). However, it should be noted that mitochondrial-targeted delivery research specifically for Sch B remains limited, and most current assumptions are extrapolated from other mitochondrial therapeutics. The feasibility, safety, and incremental benefit of these strategies within the Sch B system require systematic validation.

Regarding safety, existing animal experiments generally suggest that Sch B is tolerated under conventional doses and dosing durations, and acute and subchronic toxicity studies have not reported obvious organ toxicity or behavioral abnormalities, which is partly consistent with its historical use as an active component in traditional medicine (Yang et al., 2022). However, these findings are largely derived from short-term or moderate-dose exposure contexts and remain insufficient for comprehensive safety evaluation under high-dose, long-term, or special-population conditions. On the one hand, as a mitochondrial functional modulator, whether Sch B might interfere with energy metabolism, dynamic balance, or quality control processes in normal cells under sustained exposure lacks systematic toxicological support. On the other hand, its context-dependent bidirectional effects in tumor-related settings warrant particular caution—namely, whether Sch B may influence tumor metabolic adaptability under certain conditions while protecting normal tissues, an issue that has been preliminarily suggested in some studies (Fang et al., 2025; Yang et al., 2016). Therefore, before entering preclinical development, standardized GLP-level toxicological assessments addressing maximum tolerated dose, long-term dosing toxicity, mitochondrial functional safety boundaries, and tumor-related risks remain indispensable.

To advance Sch B from a “plant-derived metabolite with relatively clear mechanistic evidence” toward a “candidate drug with clinical evaluation value,” future research needs to deepen several key directions. First, systematic PK/PD investigations are required, not only covering different administration routes and dose levels, but also focusing on the relationship between tissue distribution and intramitochondrial exposure to improve the reliability of efficacy prediction (Wang et al., 2018). Second, dose–response relationships and potential hormesis characteristics should be systematically characterized, as existing evidence suggests that Sch B may exhibit opposite effect directions between low-dose and high-dose exposure, which directly impacts clinical dose design (Zhang et al., 2014). Third, deeper integration with modern pharmaceutics and delivery technologies is needed, including oral formulation optimization, development of long-acting preparations, and construction of mitochondria-targeted nanoplatforms (Shao et al., 2017). Fourth, cross-species and cross-disease model consistency should be validated, with large-animal models used to further evaluate stability of effects in key organs such as the heart, liver, and kidney, thereby reducing uncertainty in translation from small animals to humans (Li S. et al., 2025). Fifth, once a relatively clear safety basis is established, early clinical research pathways should be planned cautiously—for example, prioritizing perioperative organ protection, high-risk populations for DILI, or specific subgroups of chronic diseases to conduct small-sample, mechanism-oriented exploratory clinical trials (Co et al., 2024b).

Overall, Sch B possesses a clear structural foundation and has been associated with multi-organ pharmacological effects centered on mitochondrial homeostasis. However, its translational pathway is not a straightforward extension of preclinical observations and remains highly dependent on PK optimization, refinement of delivery strategies, delineation of safety boundaries, and careful selection of clinical indications. With ongoing advances in mitochondrial drug research and nanodelivery technologies, Sch B may be further evaluated in appropriate disease contexts and may contribute to the broader exploration of intervention strategies for disorders associated with mitochondrial dysfunction.

## 7 Research gaps and future directions

Although Sch B has been repeatedly reported to improve mitochondria-related functional states in multiple disease models, current understanding of its mode of action largely remains at the level of describing “mitochondria-associated effects”, rather than being grounded in a systematic theory of homeostatic regulation. Most existing studies infer mitochondrial involvement primarily from terminal phenotypes (e.g., reduced oxidative damage, improved energy status, or increased cell survival). While this approach is valuable for establishing biological relevance, it still has limitations in explaining the cross-organ consistency of Sch B. Accordingly, an unresolved question is whether the effects of Sch B in different tissues arise from parallel regulation of several independent protective pathways, or whether they involve modulation of higher-order, cross-organ shared control nodes of mitochondrial homeostasis (Nunnari and Suomalainen, 2012). Clarifying this distinction will be essential for moving beyond descriptive phenotypes toward a more unified mechanistic interpretation.

A related research gap is that current usage of the concept of “mitochondrial homeostasis” remains broad and is often equated directly with functional indices such as improved energy metabolism or antioxidant effects. However, from a systems biology perspective, maintenance of mitochondrial homeostasis also involves multiple layers, including structural integrity, functional continuity, coordinated quality control, and dynamic adaptability to environmental stress (Picard et al., 2016). Notably, although the protective effects of Sch B in the liver, kidney, heart, lung, and nervous system differ in specific manifestations, they often share a common feature of “delaying the transition from functional imbalance to irreversible injury”. This observation suggests that Sch B may not simply enhance a single mitochondrial function, but more likely influences the overall stability or resilience of the mitochondrial system under stress. At present, however, systematic quantitative and comparative analyses of such “homeostatic resilience” remain lacking, which limits deeper understanding of its unified mode of action. Future studies may benefit from developing conceptual and experimental metrics that capture resilience rather than isolated endpoints.

In addition, whether the mitochondrial regulatory effects of Sch B exhibit marked context dependence is also an important issue requiring further investigation. The major sources of mitochondrial vulnerability differ substantially across organs, including acute energy crisis, chronic metabolic load, hyperoxic exposure, or high dependence on spatial organization (Murphy and Hartley, 2018). Available studies indicate that Sch B can confer protective effects across these pathological backgrounds, but the functional emphasis and phenotypic presentation may not be fully consistent. This pattern implies that the actions of Sch B may be strongly shaped by pathological environment and metabolic state, and its regulation may resemble a “context-responsive adjustment” targeting system weak points, rather than a fixed pattern of mitochondrial functional enhancement. Currently, most studies are conducted within single-model settings, and systematic cross-model and cross-organ comparisons remain insufficient, making the above hypothesis difficult to validate. Such limitations highlight the need for more integrative experimental strategies rather than additional isolated model confirmations.

In metabolically plastic biological contexts such as the immune system and tumor system, the effects of Sch B raise additional conceptual questions. In these systems, enhanced mitochondrial function does not necessarily equate to tissue protection or functional improvement; instead, under certain conditions it may support abnormal immune activation or metabolic adaptation of tumor cells. Therefore, the mitochondrial regulatory effects of Sch B may possess bidirectional potential, and the ultimate biological outcome is highly dependent on cell type, metabolic state, and microenvironmental context. Current studies remain primarily focused on protective effects, and discussions of this potential functional tension remain relatively limited (Weinberg et al., 2015; Kenny and Birsoy, 2024). Future work should more explicitly distinguish between “homeostasis restoration” and “adaptation enhancement”, as these regulatory outcomes may carry distinct biological consequences. This distinction is particularly relevant for interpreting systemic mitochondrial modulation beyond classical injury models.

At a broader level, research on Sch B also prompts reconsideration of how mitochondria should be positioned in disease intervention. Its relatively consistent effects across multiple organ models suggest that mitochondrial homeostasis regulation may reflect a cross-tissue shared logic rather than being merely an organ- or disease-specific therapeutic target (Murphy and Hartley, 2018). However, this perspective remains largely theoretical and still lacks systematic conceptual modeling and experimental validation. Future studies integrating multi-organ models, systems-level metabolic analyses, and frameworks of inter-organ metabolic communication may help assess whether Sch B represents a broadly meaningful strategy for regulating mitochondrial homeostasis. Such efforts may also help clarify whether mitochondrial regulation should be viewed as a generalizable systemic principle rather than a collection of organ-specific observations.

Overall, current research on Sch B is gradually transitioning from the question of “whether it has mitochondria-related effects” toward “how to interpret its potential role in systemic homeostatic regulation”. The key to further advancing this field may not lie in repeatedly confirming protective phenotypes, but in more rigorously dissecting whether Sch B follows a unified homeostatic regulatory logic, whether it exhibits context-dependent regulatory features, and what diverse consequences such regulation may produce across biological systems. Continued exploration of these questions will contribute to a more comprehensive evaluation of the theoretical value of Sch B and provide a stronger conceptual basis for mitochondrial homeostasis as a systemic intervention target.

## 8 Conclusion

Based on current evidence, the pharmacological effects of Sch B across multiple organs and experimental models converge on a relatively clear common directionality: its actions are frequently associated with attenuation of mitochondrial functional imbalance or preservation of mitochondrial homeostasis, and this association is not restricted to a single disease spectrum or organ system. Unlike many natural products that are often described as acting through “multiple pathways and multiple endpoints”, research on Sch B more readily converges mechanistically on mitochondria as an upstream hub—namely, by concurrently influencing key processes including redox buffering, bioenergetic continuity, ΔΨm and mPTP threshold regulation, dynamic balance, and quality control, Sch B may reduce the likelihood that cells will cross an “irreversible injury threshold” under acute insults or chronic stress. Therefore, positioning Sch B as a “mitochondrial homeostasis–modulating plant-derived metabolite” provides a more coherent explanation for its cross-system consistency than simply emphasizing its antioxidant or anti-apoptotic properties. However, this interpretation remains primarily grounded in preclinical mechanistic synthesis rather than definitive proof of a unified mode of action.

From the perspective of mechanistic integration, existing studies suggest that mitochondrial regulation by Sch B does not rely on a single target, but rather resembles a multi-level “coordinated correction” of the mitochondrial homeostasis network. At the redox level, Sch B is frequently associated with enhancement of the glutathione system, activation of Nrf2-related antioxidant networks, and attenuation of mtDNA oxidative damage. At the energy level, Sch B is linked across multiple models to stabilization of ΔΨm, preservation of ATP-generating capacity, and reduced Ca^2+^ burden and risk of excessive mPTP opening. At the structural level, accumulating evidence supports its involvement in regulating the fusion/fission balance, while maintaining mitochondrial population quality through modulation of mitochondrial renewal and clearance processes. Importantly, autophagy/mitophagy-related evidence indicates that Sch B may exhibit pronounced context dependence: under conditions where autophagic flux is blocked or damaged mitochondria accumulate, its effects are closer to restoring quality control; whereas under backgrounds of excessive stress that may drive mitochondrial depletion, its actions may instead suppress excessive clearance to prevent energy collapse. This bidirectional regulatory pattern—rather than a simplistic “promotion” or “inhibition”—suggests that Sch B more likely modulates the “optimal range” of mitochondrial quality control to maintain cellular homeostasis. Nevertheless, many conclusions in this area remain based on marker-level inference, and standardized flux-based validation is still limited.

At the organ level, the functional emphasis of Sch B frequently aligns with the mitochondrial functional dimension that becomes limiting earliest under specific pathological conditions in different tissues. In the liver, acute toxic injury often features concurrent energy crisis and oxidative chain reactions, and Sch B commonly manifests as enhancing metabolic stress tolerance and delaying mitochondrial structural collapse. In the kidney, oxygen gradients and perfusion fluctuations make I/R-associated “abrupt” mitochondrial imbalance more prominent, and Sch B effects are more readily reflected by improved tolerance to redox fluctuation and enhanced dynamic stability. In the heart, extreme high energy flux makes ΔΨm and the mPTP threshold highly sensitive, and Sch B-related findings commonly align with mitochondrial stabilization during early reperfusion and an upward shift of the apoptotic threshold. In the nervous system, vulnerability is driven by precision of energy supply, spatial distribution, and transport dependence, and protective phenotypes are more often associated with maintenance of long-term mitochondrial homeostasis, synaptic energy support, and reduced accumulation of chronic injury. In the lung, chronic hyperoxic exposure and inflammatory pressure emphasize oxidative burden and barrier energy maintenance, and Sch B effects are more likely reflected by reduction of injury amplification loops and preservation of minimal energy output. The tumor system represents a distinct scenario in which mitochondrial meaning is partially inverted: mitochondrial homeostasis in tumor cells often supports stress adaptation and therapeutic tolerance, and therefore the pro-apoptotic or sensitizing effects of Sch B observed in some models may derive from compression of tumor mitochondrial adaptive reserve. This also highlights that systemic applications of Sch B must simultaneously consider differential impacts on normal tissues and tumor tissues. Such context dependence further complicates efforts to generalize organ-protective conclusions across disease settings.

Nevertheless, the strength and translational potential of the cross-model evidence for Sch B should be interpreted more cautiously. First, the existing literature is highly heterogeneous, with substantial differences in dose, treatment duration, model severity, species, and endpoint measurements across studies, such that the boundary between “reproducible mechanistic axes” and “model-specific phenomena” still requires more systematic comparative research. Second, the most critical translational issue—effective intramitochondrial exposure—remains supported mainly by indirect evidence, because plasma and total tissue concentrations cannot be directly equated with free intramitochondrial concentrations, retention time, or targeting efficiency. For a candidate molecule positioned as a mitochondrial homeostasis modulator, subcellular-level PK/PD modeling, systematic characterization of dose–effect and time-window patterns, and rigorous validation of potential toxicity will directly determine whether Sch B can move from being “mechanistically plausible” to “developable”. In addition, studies showing pro-apoptotic or cytotoxicity-related signals under certain conditions suggest that safety boundaries, long-term exposure effects, and interindividual variability (especially sex differences) require more standardized toxicology and risk assessment frameworks, to avoid extrapolating “multi-organ protection” into assumptions of universal safety.

Overall, this review uses mitochondrial homeostasis as the central axis to converge mechanistic evidence and explain organ-specific differences in the multi-organ effects of Sch B, and proposes a “unified mechanistic model” as an integrative framework spanning multi-system evidence. The value of this framework lies in that it not only synthesizes existing findings but also provides testable hypotheses for future research—namely, by applying stricter mitochondrial functional readouts, more comparable cross-model designs, and subcellular exposure–effect correlations, to evaluate whether Sch B indeed represents a mitochondrial homeostasis–modulating strategy of systemic significance. If future work can generate stronger evidence in delivery optimization, intramitochondrial exposure quantification, safety boundary definition, and indication positioning, Sch B may further gain translational evaluation value in clinically relevant contexts such as liver fibrosis, I/R-related organ injury, and metabolic chronic diseases. However, such advancement will ultimately depend on rigorous mechanistic validation and carefully designed translational studies rather than extension of preclinical associations alone.
